# Supplementary material for: Differential PROTAC substrate specificity dictated by orientation of recruited E3 ligase
Source: Nat Commun. 2019 Jan 10;10:131. doi: 10.1038/s41467-018-08027-7 (PMC6328587; doi:10.1038/s41467-018-08027-7)
Supplement: Supplementary file 1 — Supplementary Information [file 41467_2018_8027_MOESM1_ESM.pdf]

Supplementary Information

Differential PROTAC Substrate Specificity Dictated by Orientation of Recruited E3 Ligase

Smith et al.

Supplementary Figure 1

Linker length (amide series)

SJF-6693

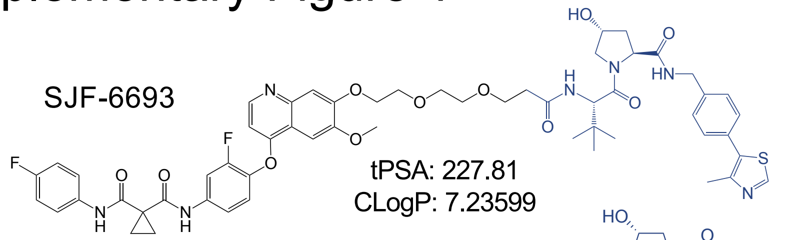

tPSA: 227.81
CLogP: 7.23599

10 atoms

SJF-6690

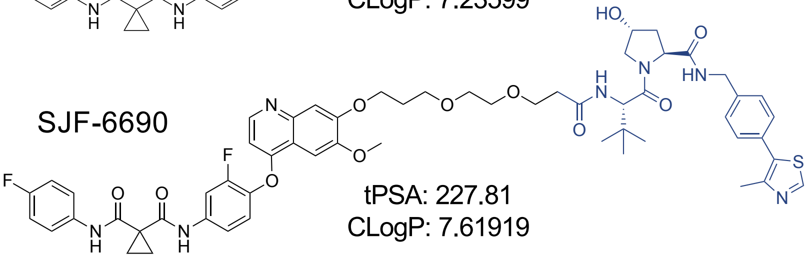

tPSA: 227.81
CLogP: 7.61919

11 atoms

SJF-8240

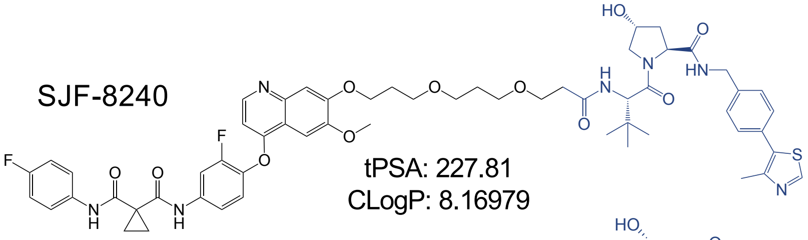

tPSA: 227.81
CLogP: 8.16979

12 atoms

SJFα
(SJF-6696)

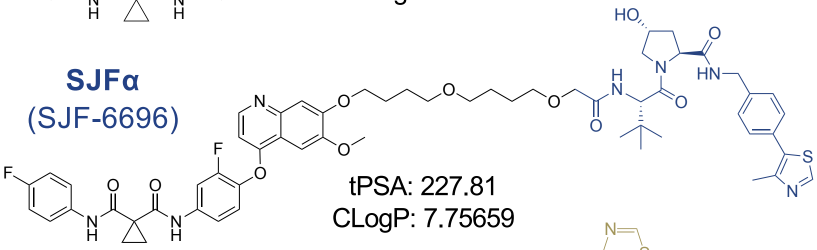

tPSA: 227.81
CLogP: 7.75659

13 atoms

Linker length (phenyl series)

SJFδ
(SJF-6683)

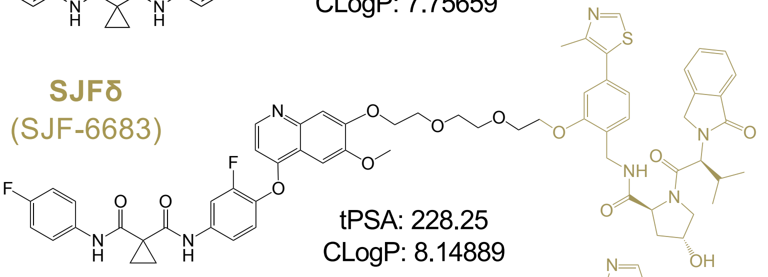

tPSA: 228.25
CLogP: 8.14889

10 atoms

SJF-6677

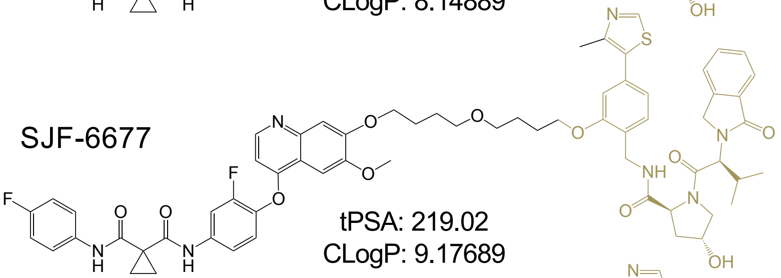

tPSA: 219.02
CLogP: 9.17689

11 atoms

BES-4612

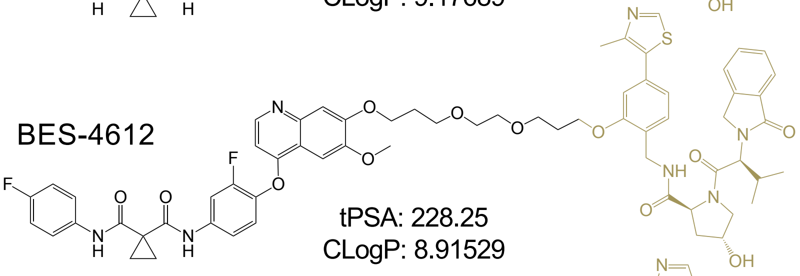

tPSA: 228.25
CLogP: 8.91529

12 atoms

SJF-6685

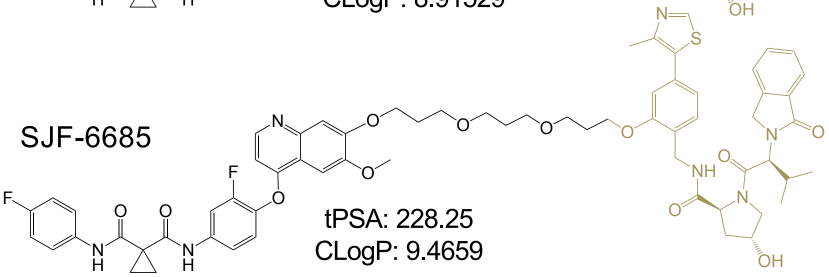

tPSA: 228.25
CLogP: 9.4659

13 atoms

Supplementary Figure 1. PROTAC structures (related to Figures 1-6). Structures of all PROTACs used in this study with the calculated total polar surface area (tPSA) and CLogP values for each structure. The eight PROTACs are divided into two groups – “amide series” and “phenyl series” – based on the differences in their VHL-recruiting moieties (colored in dark blue and gold, respectively). Each series contains four PROTACs based on a foretinib warhead and linker lengths of 10, 11, 12, and 13 atoms. The two lead PROTACs (SJF-6696 and SJF-6683) used throughout the study are also highlighted in Figure 1d.

Supplementary Figure 2

a

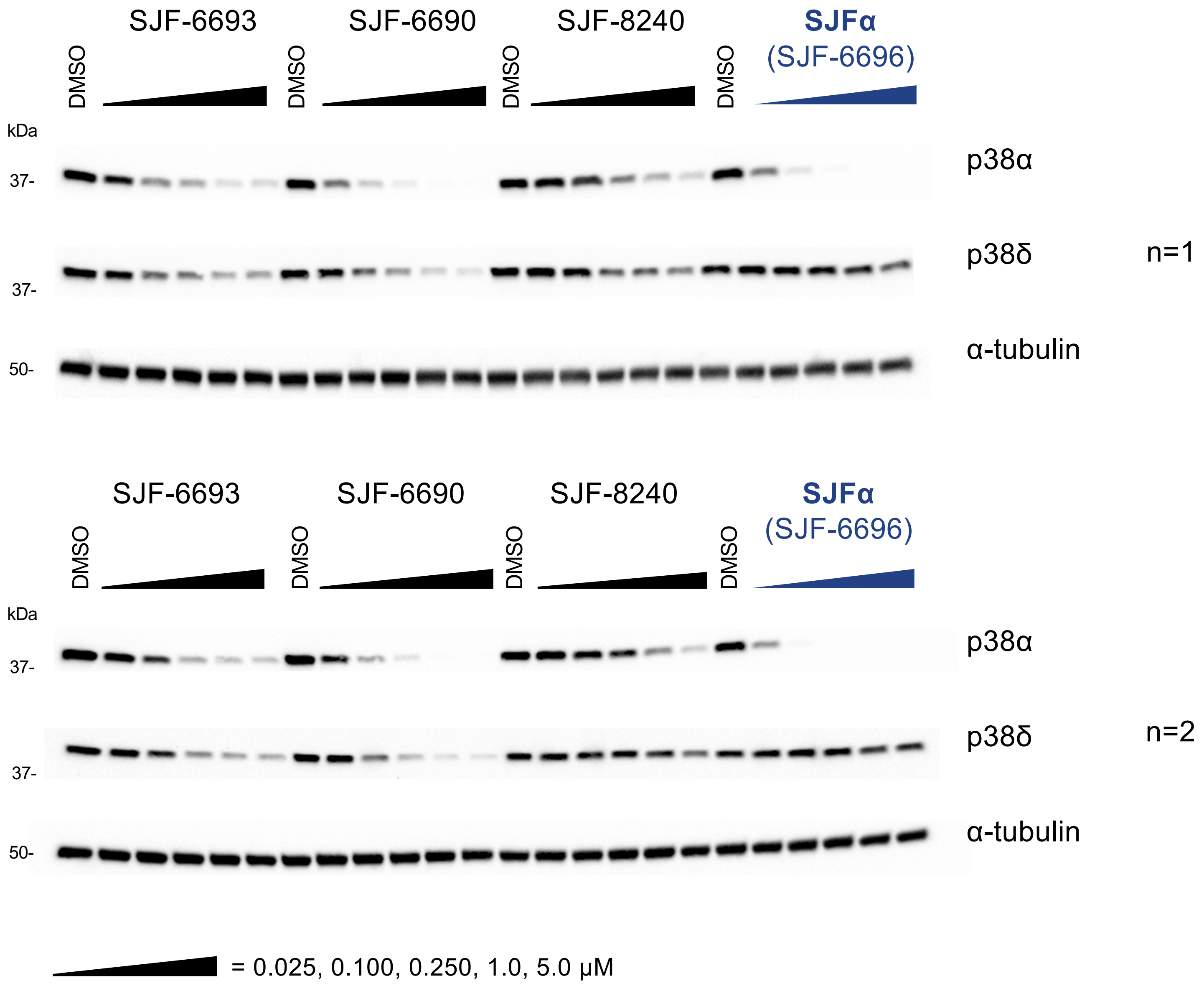

Supplementary Figure 2 cont.

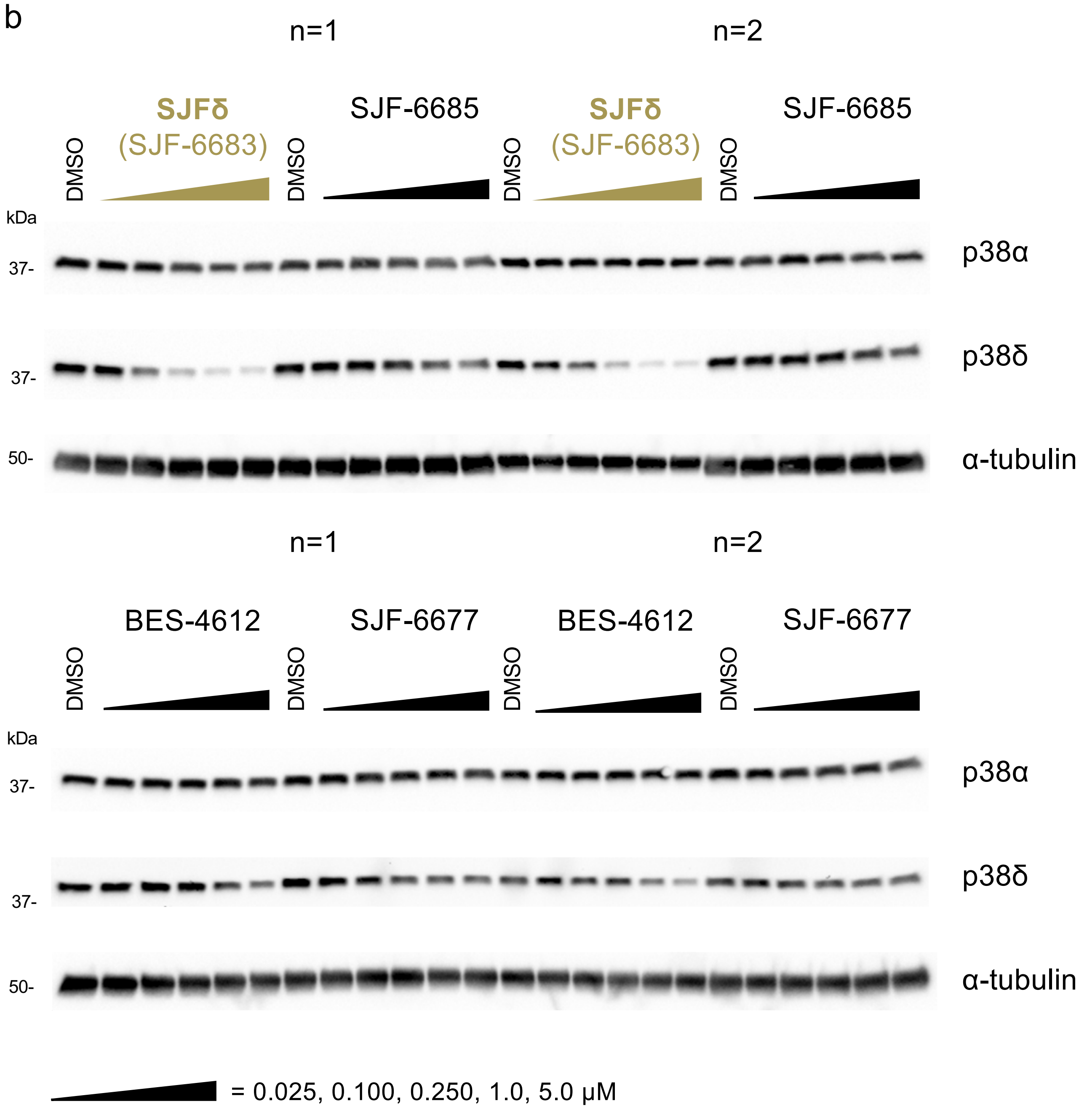

Supplementary Figure 2. Survey of p38 isoform degradation with foretinib-based VHL PROTACs (related to Figure 1 and Supplementary Table 1). (a) Western blots of amide series PROTACs tested in increasing concentrations (0.025, 0.100, 0.250, 1.0, 5.0 μM) on MDA-MB-231 cells, in duplicate. Amide series lead PROTAC SJF-6696 (referred to as SJFα) is highlighted in dark blue. **(b)** Same as in (a) but with the phenyl series PROTACs. Phenyl series lead PROTAC SJF-6683 (referred to as SJFδ) is highlighted in gold. Summary table of the DC_{50} (concentration at which half-maximal degradation is achieved) and D_{max} (maximum percentage of degradation achieved) is reported, per PROTAC, in Supplementary Table 1. Data is based on biological duplicates and is normalized to α-tubulin.

Supplementary Table 1

Summary of p38α/p38δ degradation*		
**Amide PROTAC series**	p38α	p38δ
SJF-6693 (10 atoms)	DC_{50} = 40.1 ± 7.7 nM<br>D_{max} = 92.6 ± 2.8%	DC_{50} = 51.4 ± 7.4 nM<br>D_{max} = 81.8 ± 1.9%
SJF-6690 (11 atoms)	DC_{50} = 18.8 ± 2.7 nM<br>D_{max} = 99.4 ± 2.0%	DC_{50} = 91.7 ± 18 nM<br>D_{max} = 96.7 ± 3.7%
SJF-8240 (12 atoms)	DC_{50} = 230 ± 47 nM<br>D_{max} = 90.1 ± 4.2%	DC_{50} = 456 ± 362 nM<br>D_{max} = 54.3 ± 11.8%
**SJFα** (SJF-6696, 13 atoms)	DC_{50} = 9.5 ± 1.0 nM<br>D_{max} = 99.6 ± 1.1%	DC_{50} = 1.16 ± 2.47 μM<br>D_{max} = 25.1 ± 22.7%
**Phenyl PROTAC series**	p38α	p38δ
**SJFδ** (SJF-6683, 10 atoms)	DC_{50} = 45.9 ± 81.3 nM<br>D_{max} = 34.5 ± 10.1%	DC_{50} = 79.2 ± 28 nM<br>D_{max} = 98.4 ± 6.6%
SJF-6677 (11 atoms)	DC_{50} = 127 ± 163 nM<br>D_{max} = 25.3 ± 6.6%	DC_{50} = 106 ± 81.5 nM<br>D_{max} = 43.5 ± 7.2%
BES-4612 (12 atoms)	DC_{50} = 0.76 ± 1.5 μM<br>D_{max} = 16.8 ± 13.4%	DC_{50} = 1.89 ± 2.35 μM<br>D_{max} = 78.7 ± 40.8%
SJF-6685 (13 atoms)	DC_{50} = 304 ± 691 nM<br>D_{max} = 19.9 ± 11.3%	DC_{50} = 1.14 ± 1.95 μM<br>D_{max} = 33.5 ± 27.2%

*These +/- values represent one standard deviation from the mean of the calculated DC_{50} or D_{max} value.

Supplementary Figure 3

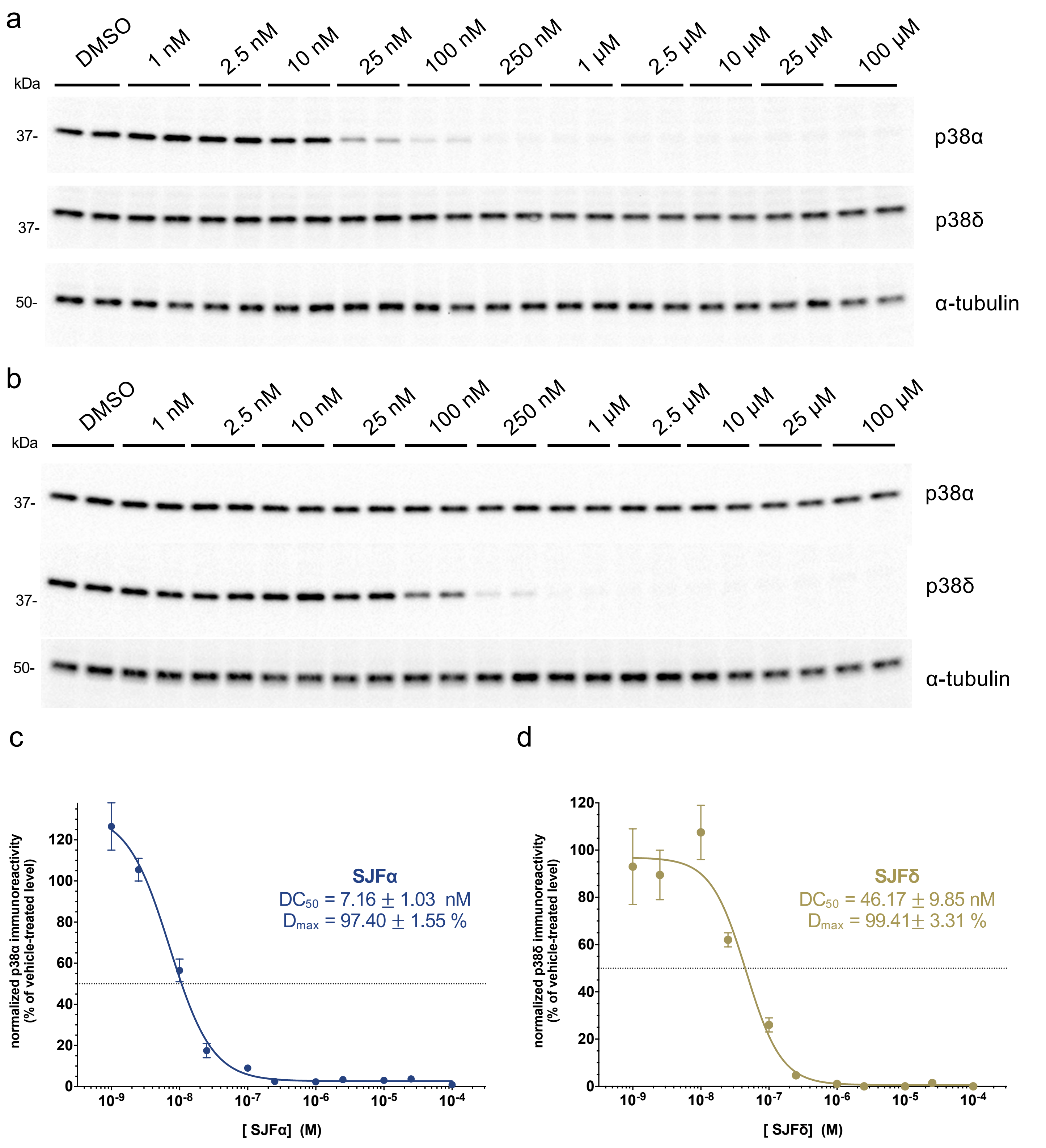

Supplementary Figure 3. Foretinib-based PROTACs selectively degrade different isoforms of p38 MAPK through differential orientation of recruited VHL (related to Figure 1). Western blots represent a wider dose response to test for hook effect. **(a)** Dose-dependent and selective degradation of p38α by foretinib-based PROTAC SJFα with "left-handed" amide linkage to VHL ligand. **(b)** Dose-dependent and selective degradation of p38δ by foretinib-based PROTAC SJFδ with "right-handed" phenyl linkage to VHL ligand. **(c)** Quantitation of normalized p38α levels from panel (a). (**d**) Quantitation of normalized p38δ levels from panel (b). Levels of p38 are normalized to α-tubulin and values expressed relative to those from cells treated with DMSO (vehicle). Error bars display the s.d. of duplicate experiments.

Supplementary Figure 4

a

```
p38alpha    1 MSQE---RPTFYRQELNKTIWEVPERYQNLSPVGSGAYGSVCAAFDTKTGLRVAVKKLSRPFQSIIHAKRTYRE  71
p38beta     1 MSGP---RAGFYRQELNKTVWEVPQRLQGLRPVGSGAYGSVCSAYDARLRQKVAVKKLSRPFQSLIHARRTYRE  71
p38delta    1 MSLIR--KKGFYKQDVNKTAWELPKTYVSPTHVGSGAYGSVCSAIDKRSGEKVAIKKLSRPFQSEIFAKRAYRE  72
p38gamma    1 MSSPPPARSGFYRQEVTKTAWEVRAVYRDLQPVGSGAYGAVCSAVDGRTGAKVAIKKLYRPFQSELFAKRAYRE  74

p38alpha   72 LRLLKHMKHENVIGLLDVFTPARSLEEFNDVYLVTHLMGADLNNIVKCQKLTDDHVQFLIYQILRGLKYIHSAD 145
p38beta    72 LRLLKHLKHENVIGLLDVFTPATSIEDFSEVYLVTTLMGADLNNIVKCQALSDEHVQFLVYQLLRGLKYIHSAG 145
p38delta   73 LLLLKHMQHENVIGLLDVFTPASSLRNFYDFYLVMPFMQTDLQKIMGM-EFSEEKIQYLVYQMLKGLKYIHSAG 145
p38gamma   75 LRLLKHMRHENVIGLLDVFTPDETLDDFTDFYLVMPFMGTDLGKLMKHEKLGEDRIQFLVYQMLKGLRYIHAAG 148

p38alpha  146 IIHRDLKPSNLAVNEDCELKILDFGLARHTDDEMTGYVATRWYRAPEIMLNWMHYNQTVDIWSVGCIMAELLTG 219
p38beta   146 IIHRDLKPSNVAVNEDCELRILDFGLARQADEEMTGYVATRWYRAPEIMLNWMHYNQTVDIWSVGCIMAELLQG 219
p38delta  146 VVHRDLKPGNLAVNEDCELKILDFGLARHADAEMTGYVVTRWYRAPEVILSWMHYNQTVDIWSVGCIMAEMLTG 219
p38gamma  149 IIHRDLKPGNLAVNEDCELKILDFGLARQADSEMTGYVVTRWYRAPEVILNWMRYTQTVDIWSVGCIMAEMITG 222

p38alpha  220 RTLFPGTDHINQLQQIMRLTGTPPAYLINRMPSHEARNYIQSLTQMPKMNFANVFIGANPLAVDLLEKMLVLDS 293
p38beta   220 KALFPGSDYIDQLKRIMEVVGTPSPEVLAKISSEHARTYIQSLPPMPQKDLSSIFRGANPLAIDLLGRMLVLDS 293
p38delta  220 KTLFKGKDYLDQLTQILKVTGVPGTEFVQKLNDKAAKSYIQSLPQTPRKDFTQLFPRASPQAADLLEKMLELDV 293
p38gamma  223 KTLFKGSDHLDQLKEIMKVTGTPPAEFVQRLQSDEAKNYMKGLPELEKKDFASILTNASPLAVNLLEKMLVLDA 296

p38alpha  294 DKRITAAQALAHAYFAQYHDPDDEPVA-DPYDQSFESRDLLIDEWKSLTYDEVISFVPPPLDQE--EME--S   360
p38beta   294 DQRVSAAEALAHAYFSQYHDPEDEPEA-EPYDESVEAKERTLEEWKELTYQEVLSFKPPEPPKPPGSLEIEQ   364
p38delta  294 DKRLTAAQALTHPFFEPFRDPEEETEAQQPFDDSLEHEKLTVDEWKQHIYKEIVNFSPIARKDSRRRSGMKL   365
p38gamma  297 EQRVTAGEALAHPYFESLHDTEDEPQV-QKYDDSFDDVDRTLDEWKRVTYKEVLSFKPPRQLGARVSKETPL   367
```

b

Summary of p38 MAPK alignments				
	**p38α** sequence alignment	**p38α** r.m.s.d	**p38δ** sequence alignment	**p38δ** r.m.s.d
**p38α** (360 aa)	100%	100%	60.71%	1.7 Å (over 336 residues)
**p38β** (364 aa)	72.34%	-	60.35%	-
**p38δ** (365 aa)	60.71%	1.7 Å (over 336 residues)	100%	100%
**p38γ** (367 aa)	58.97%	2.4 Å (over 312 residues)	67.15%	1.8 Å (over 304 residues)

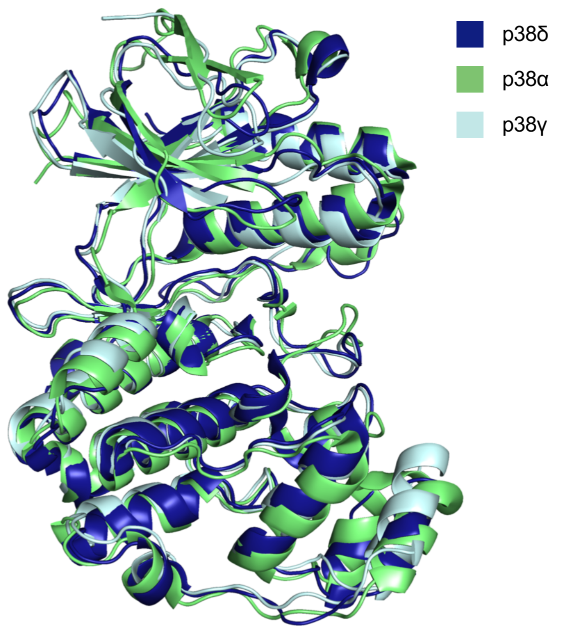

Supplementary Figure 4 cont.

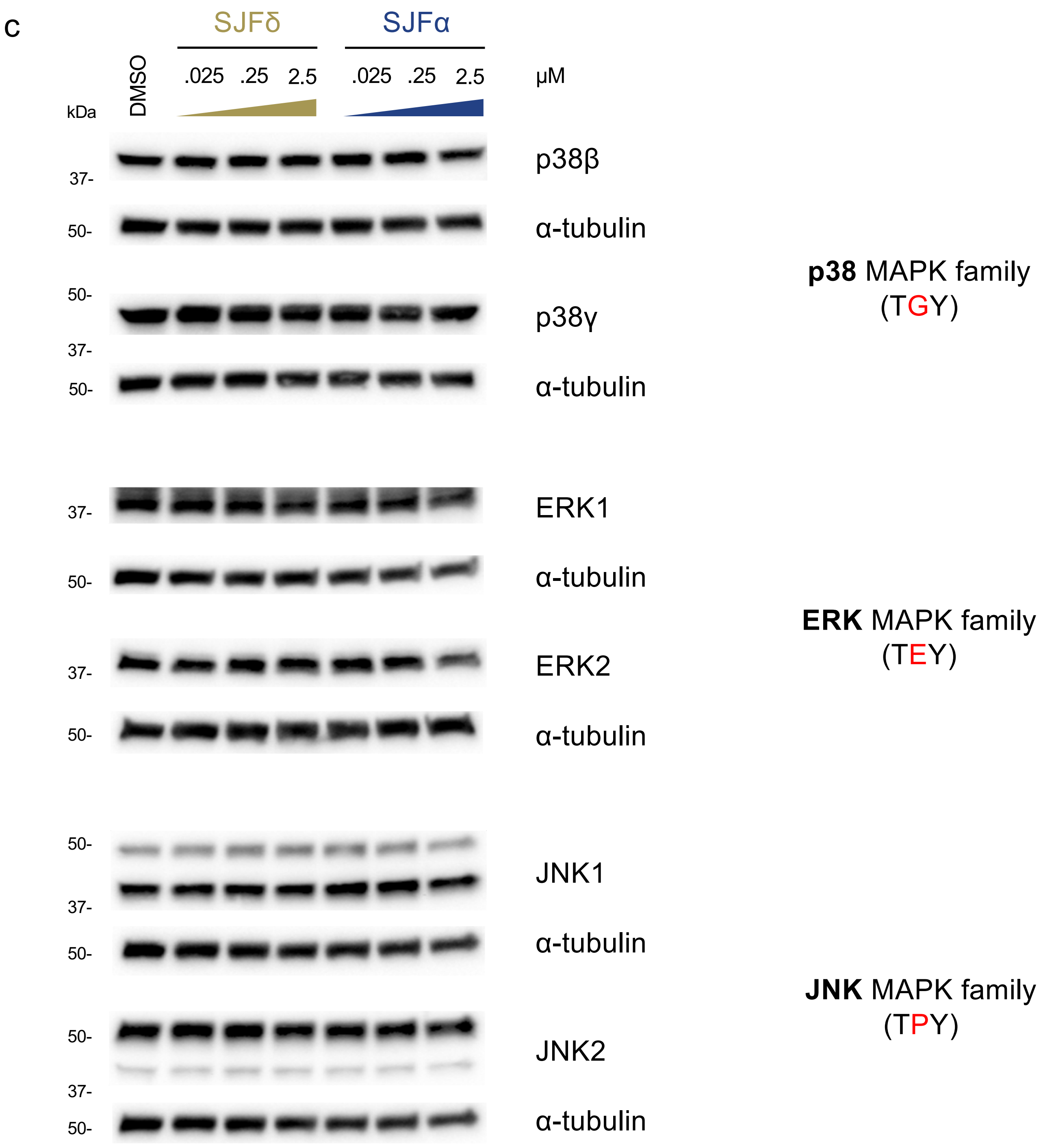

Supplementary Figure 4. Characterization of MAPK family degradation selectivity (related to Figures 1-2). (a) p38 MAPK family members (α, β, δ, γ) were aligned using the T-Coffee multiple sequence alignment server. Output results were then colored according to conservation (dark turquoise) and identity (lighter shades of turquoise) in Jalview. **(b)** Table summarizing pairwise sequence alignments (obtained in Jalview) and structural alignments for Cα atoms (obtained in PyMOL) using the doubly-phosphorylated (pTGpY) active conformations of p38δ (PDB: 4MYG), p38α (PDB: 3PY3), and p38γ (PDB: 1CM8). No active conformation crystal structure of p38β currently exists. **(c)** Western blots characterizing the lack of degradation of the remaining p38 MAPK (p38β, p38γ), ERK MAPK (ERK1, ERK2), and JNK MAPK (JNK1, JNK2) family members with increasing concentrations of SJFα and SJFδ in MDA-MB-231 cells. MAPK families are also divided based on their phosphorylation lip motif, (pTXpY), highlighted in red. Note: JNK1 and JNK2 are expressed as two alternatively-spliced forms. JNK3 was not expressed in these cells.

Supplementary Figure 5

a

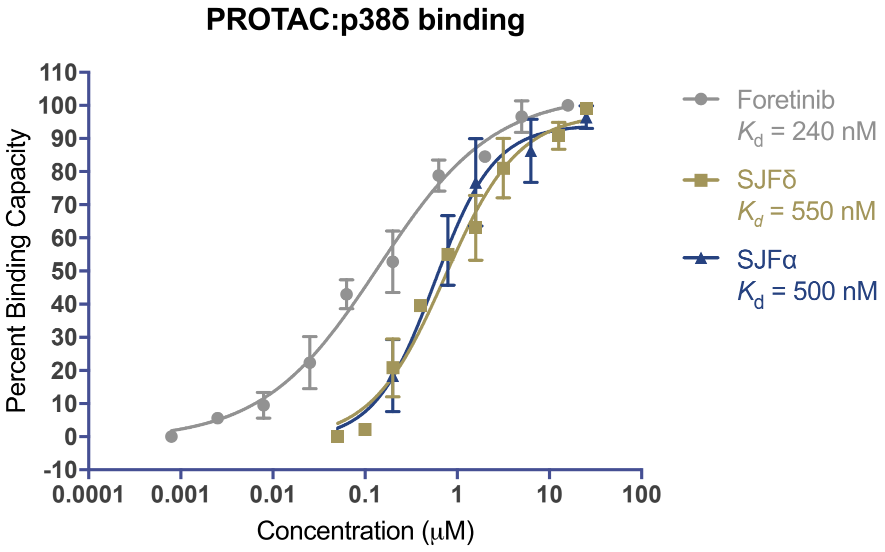

b

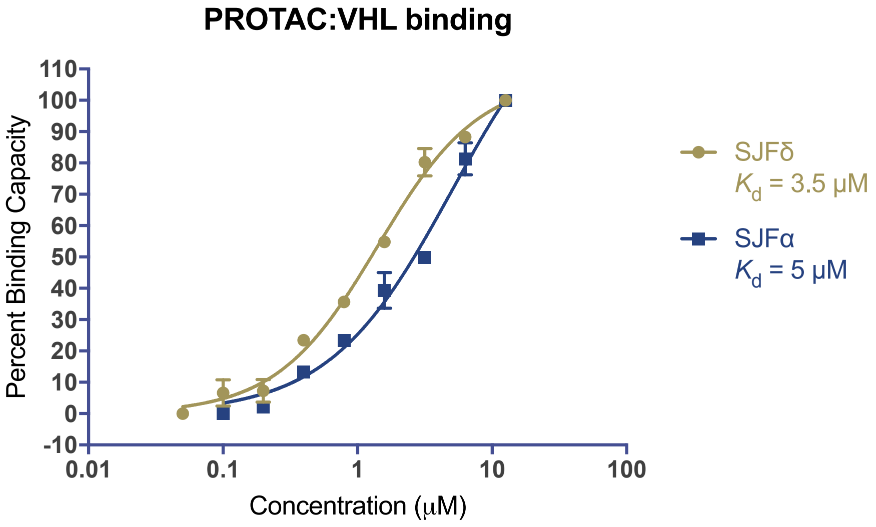

Supplementary Figure 5 cont.

c

Replicate 1
p38δ:SJFδ:VHL binding (K_d): 358 nM

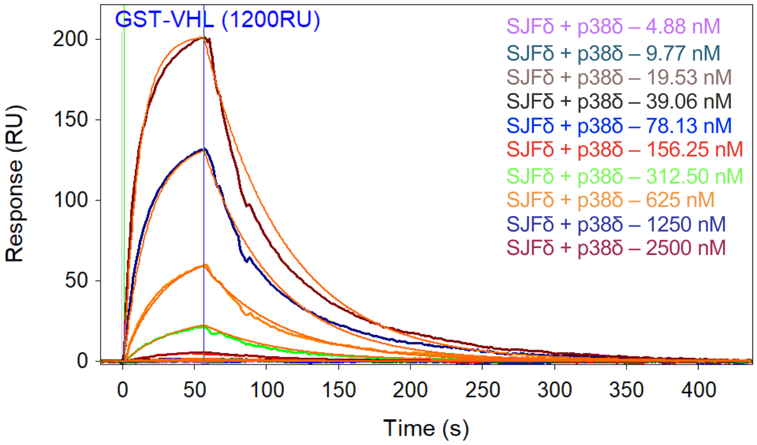

k_{on} = 5.02 x 10^4 M^{-1} s^{-1}
k_{off} = 0.018 s^{-1}
$t_{1/2}$ = 39 s

Replicate 2
p38δ:SJFδ:VHL binding (K_d): 514 nM

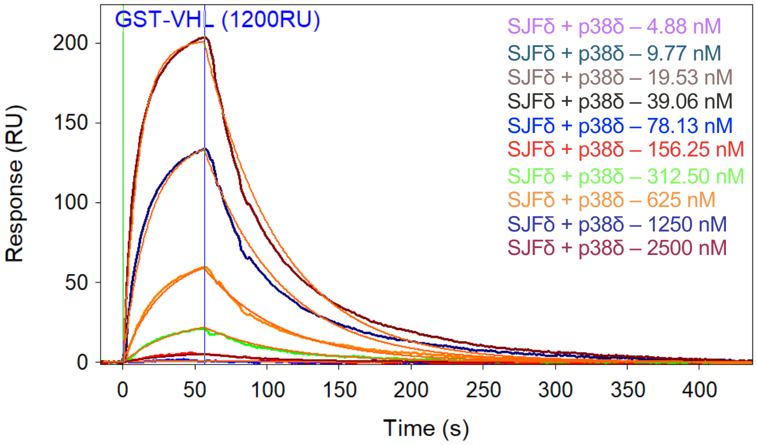

k_{on} = 2.91 x 10^4 M^{-1} s^{-1}
k_{off} = 0.017 s^{-1}
$t_{1/2}$ = 37 s

Replicate 1
p38δ:SJFα:VHL binding (K_d): 1200 nM

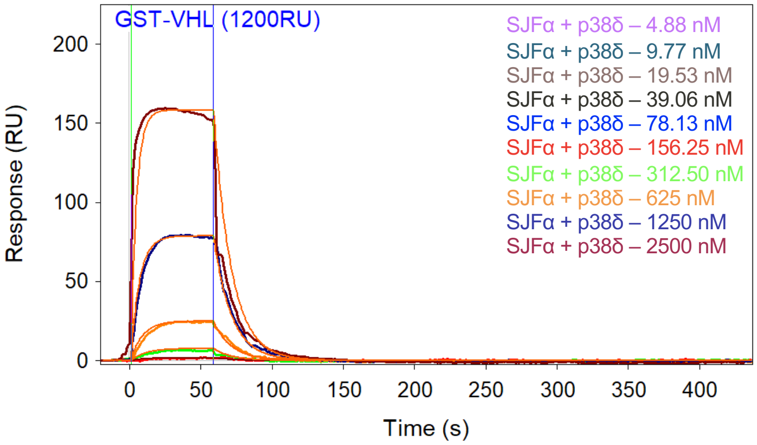

k_{on} = 7.01 x 10^4 M^{-1} s^{-1}
k_{off} = 0.083 s^{-1}
$t_{1/2}$ = 8 s

Replicate 2
p38δ:SJFα:VHL binding (K_d): 1200 nM

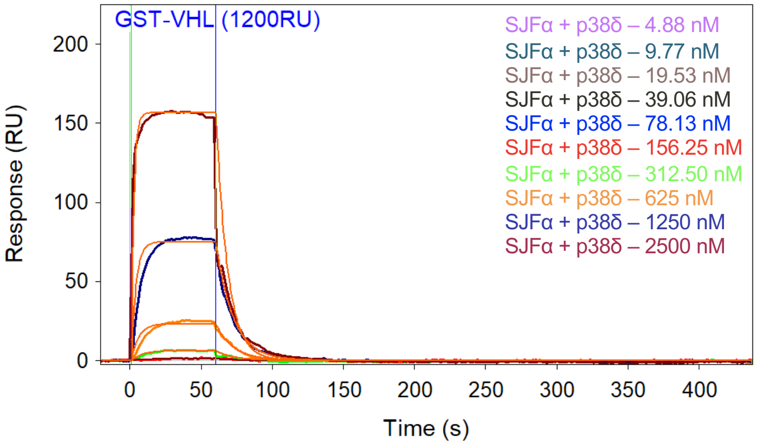

k_{on} = 7.01 x 10^4 M^{-1} s^{-1}
k_{off} = 0.082 s^{-1}
$t_{1/2}$ = 8.5 s

Supplementary Figure 5. SPR curves assessing binary and ternary interactions (related to Figure 4 and Table 1). (a) Surface plasmon resonance (SPR) was used to measure the affinity of interaction (K_d) between His-p38δ and compound (foretinib, SJFδ, or SJFα) and **(b)** GST-VBC and PROTAC (SJFδ or SJFα). Error bars display the s.d. of duplicate experiments. **(c)** SPR kinetic evaluation of p38δ:SJFδ:VHL and p38δ:SJFα:VHL ternary complex interactions. Equimolar p38δ:PROTAC mixtures were injected onto immobilized GST-VBC and allowed to dissociate over time. The p38δ:SJFδ:VHL ternary affinity is greater than the p38δ:SJFα:VHL ternary affinity owed, in part, to the slower k_{off} of the former. See Table 1 for a summary of the binary and ternary affinity and kinetic measurements.

Supplementary Figure 6

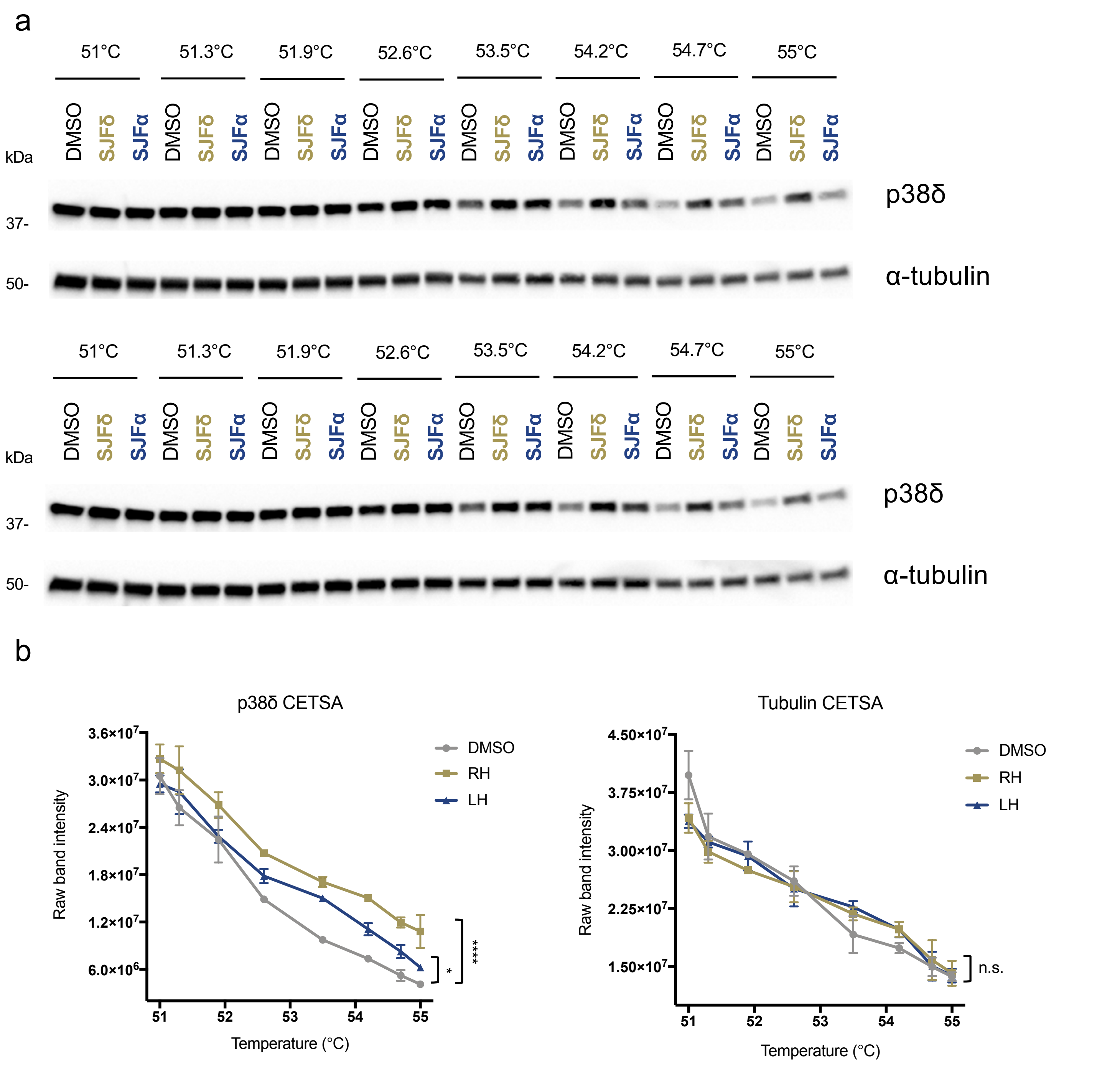

Supplementary Figure 6. CETSA reveals significant SJFα- and SJFδ-induced p38δ thermal shifts (related to Figure 4). (a) Cellular thermal shift assay (CETSA) was conducted with MDA-MB-231 cell lysate as a means to monitor cellular target engagement. Cell lysate was incubated with either vehicle (DMSO), SJFα (100 μM), or SJFδ (100 μM) for 30 minutes prior to melting at the indicated temperatures. Assay was performed in duplicate and western blots of p38δ and α-tubulin (negative control) are shown. **(b)** Raw band intensities from (a) were measured, averaged, and reported (with s.d.) with increasing temperature (51-55°C) from left-to-right. Top: p38δ CETSA analysis. Student's t-tests were performed between DMSO and SJFδ and DMSO and SJFα, revealing significant thermal shifts with SJFδ (****$p<0.0001$) and SJFα (*$p=0.0121$). Bottom: α-tubulin CETSA analysis. Student's t-tests were performed between DMSO and SJFδ and DMSO and SJFα, revealing no significant thermal shifts with either compound. n.s. = not significant.

Supplementary Figure 7

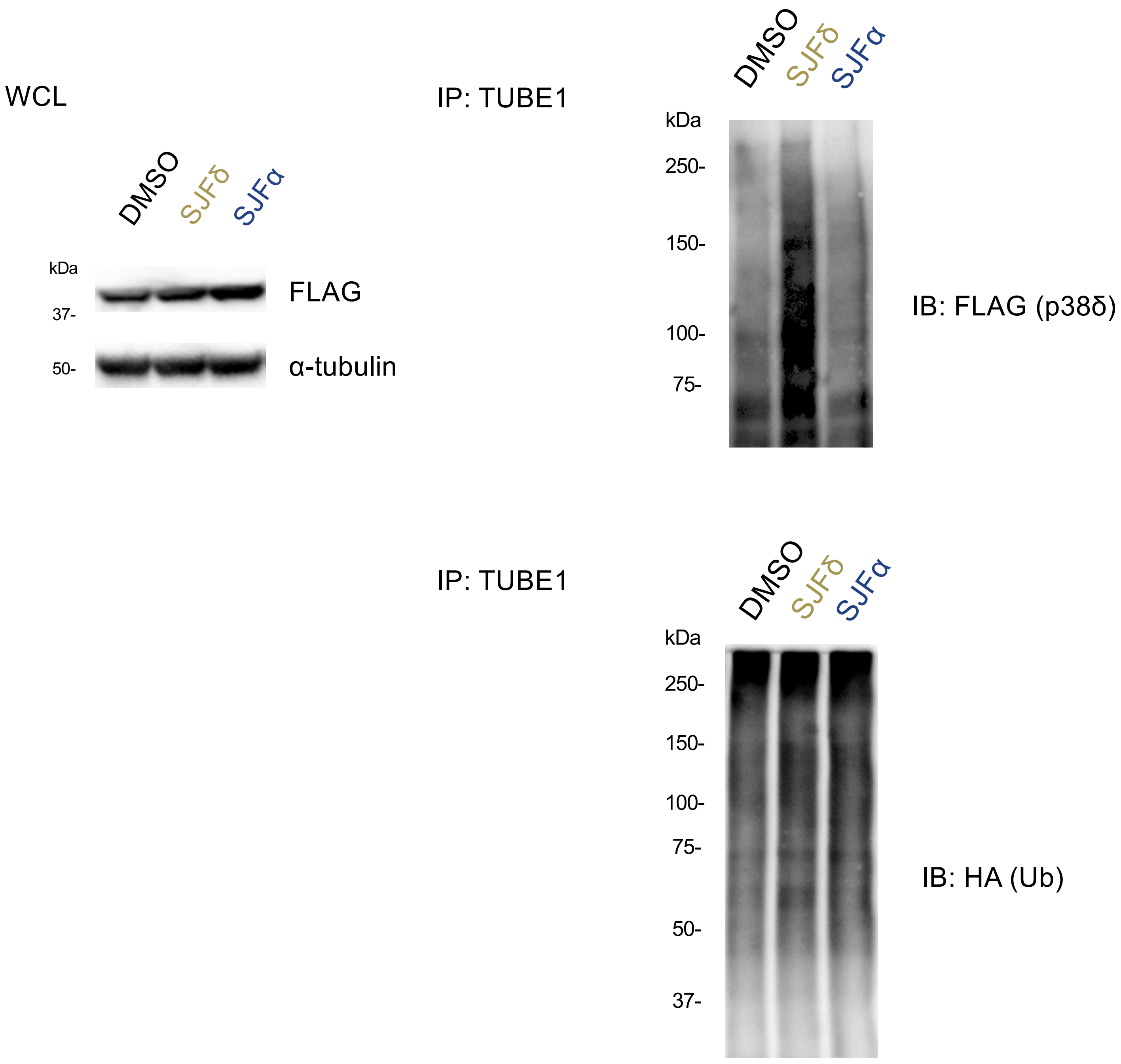

Supplementary Figure 7. SJFδ induces polyubiquitination of p38δ (related to Figure 4). HeLa cells were co-transfected with HA-Ub and FLAG p38δ and treated with vehicle (DMSO), 1 μM SJFα, or 1 μM SJFδ for 3 hours. Lysates were immunoprecipitated with tandem ubiquitin binding entity (TUBE1) agarose beads and assessed by western blots detecting FLAG (p38δ). TUBE1 binds tetra ubiquitin chains with high affinity, thereby enriching for polyubiquitinated proteins. In this assay, HA (Ub) serves as an immunoprecipitation control for poly-Ub TUBE1 enrichment.

Supplementary Figure 8

Figure 1c

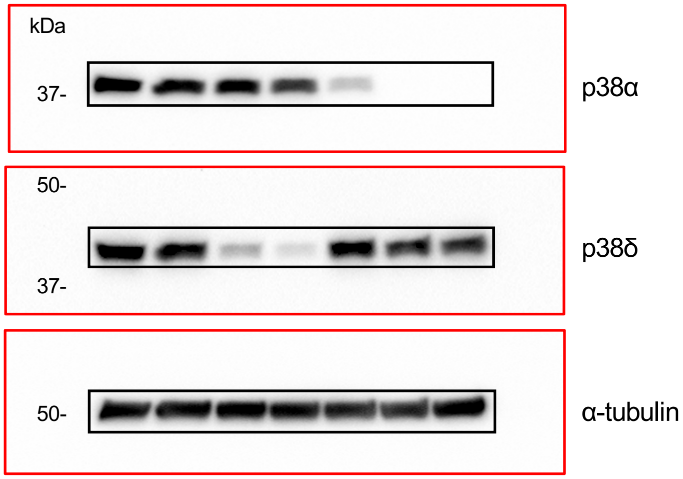

Figure 2a

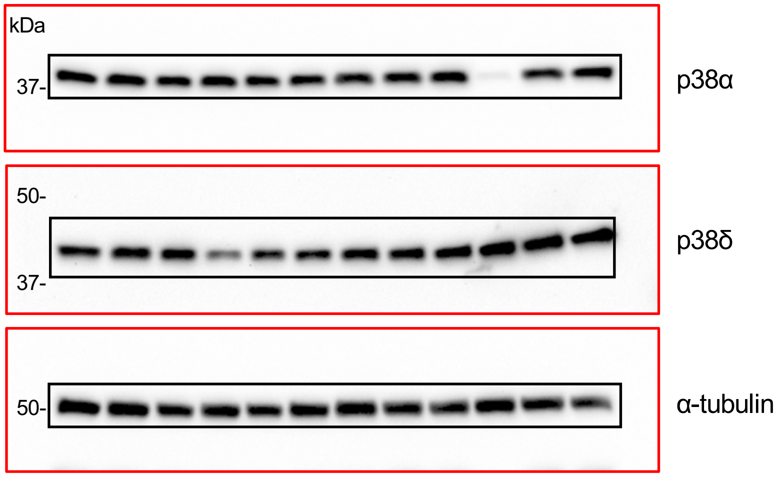

Figure 2c

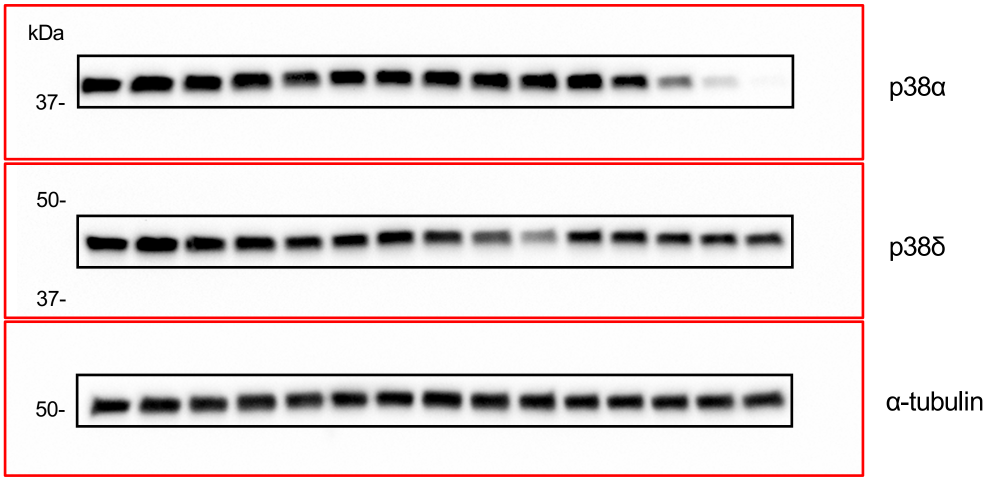

Figure 2d

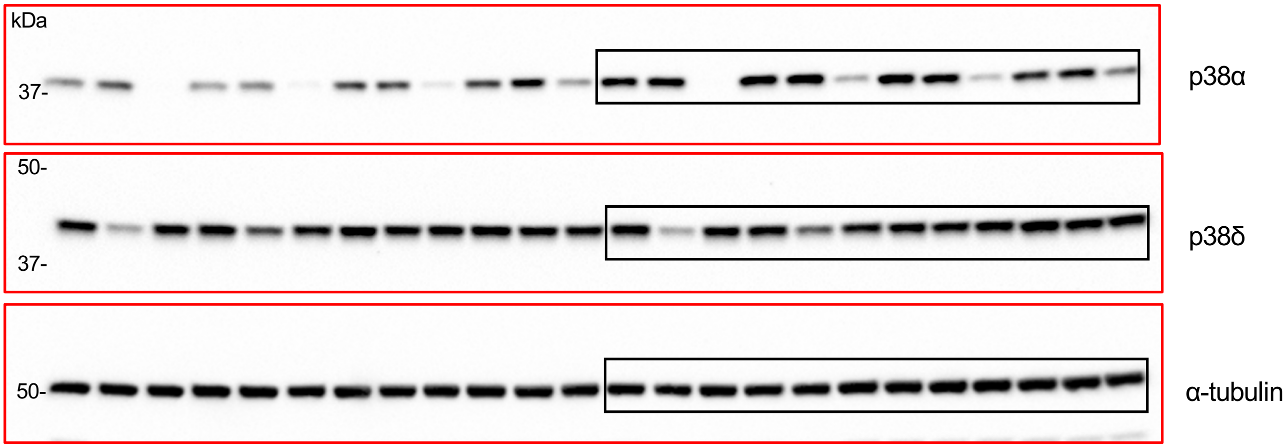

Figure 3a

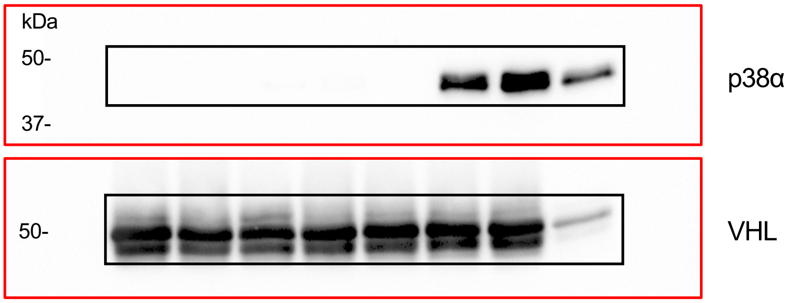

Supplementary Figure 8

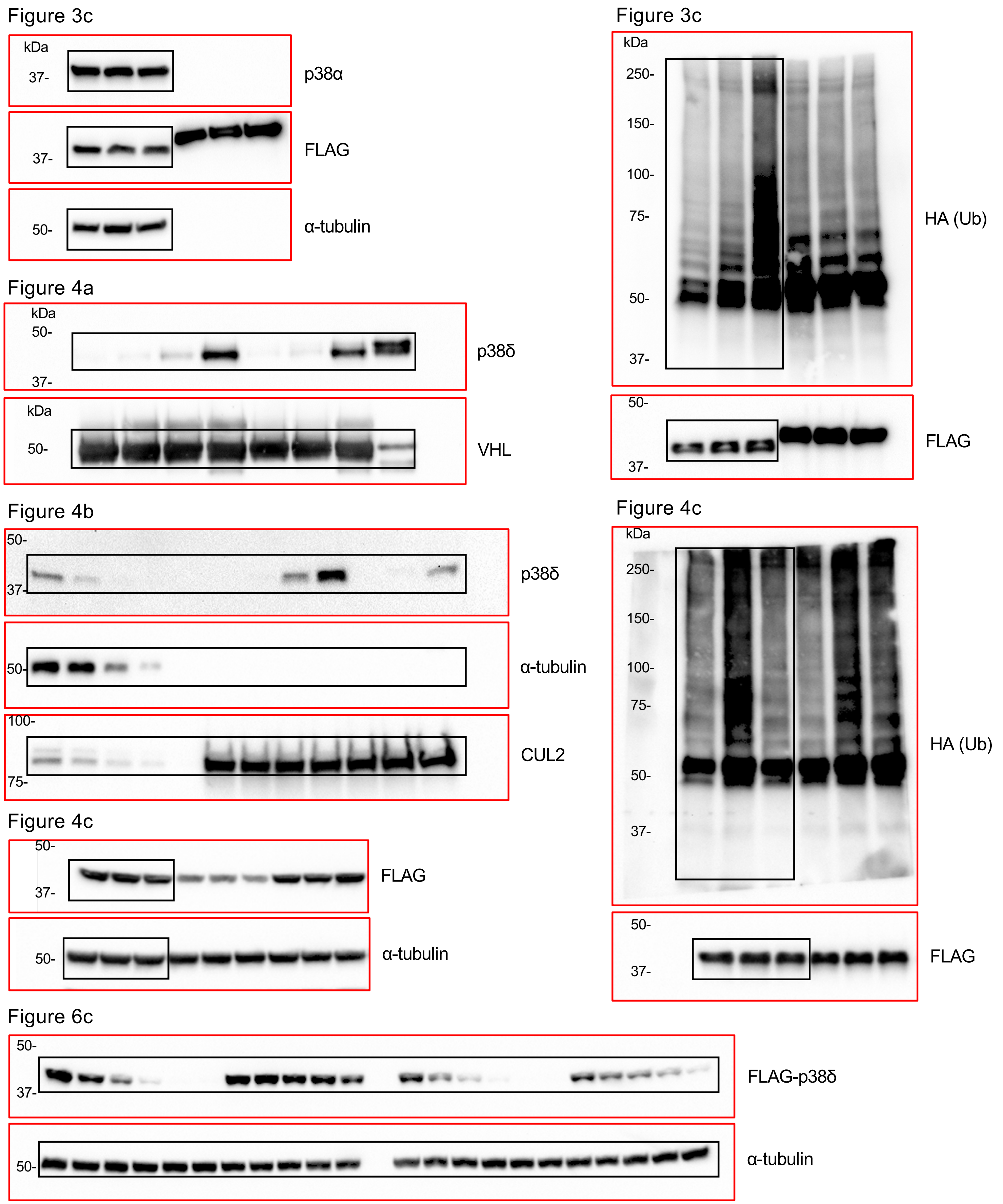

Supplementary Figure 8. Uncropped images of western blots displayed in the manuscript. Red outline demarcates the uncropped western blot, whereas the black outline indicates the cropped image.

Supplementary Note 1
Chemical syntheses of PROTAC compounds

Scheme 1.- Synthesis of PROTAC **SJF-6677**.

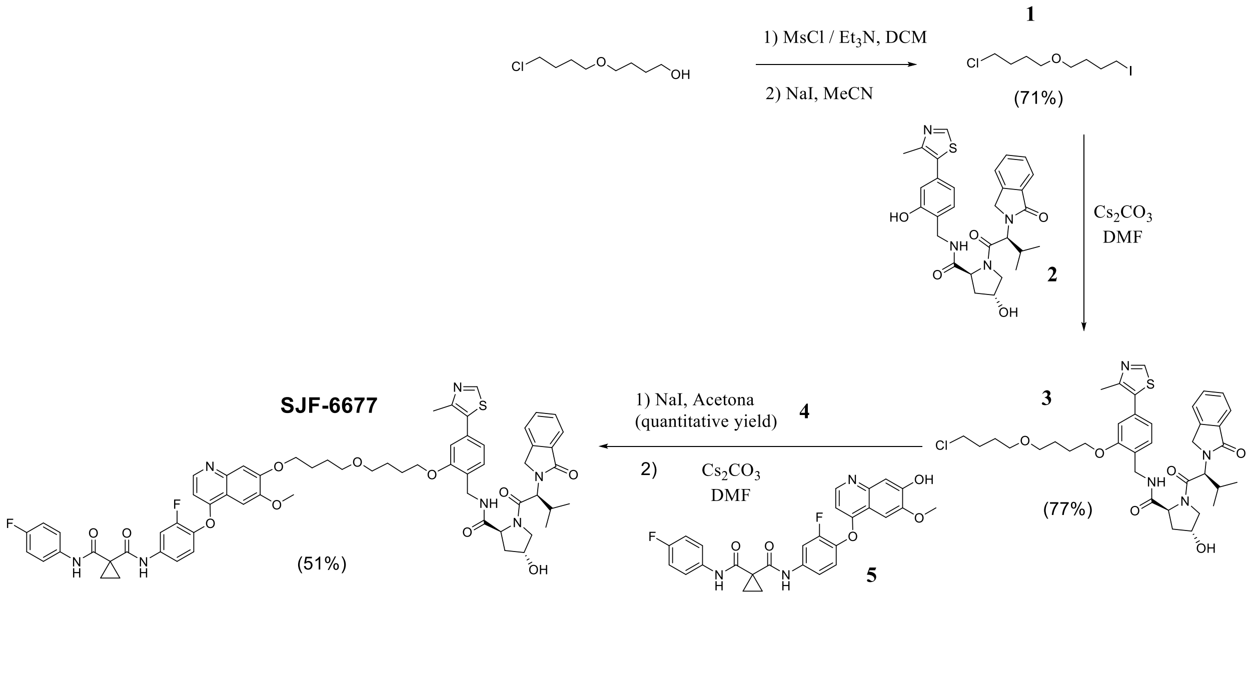

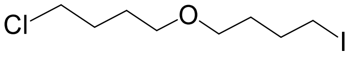

1-chloro-4-(4-iodobutoxy)butane (1). To a solution of 4-(4-chlorobutoxy)butan-1-ol (271 mg, 1.5 mmol) in Dichloromethane (5 ml) was added TEA (0.63 ml, 4.5 mmol), then reaction mixture was cooled to 0 ^{o}C (water ice/acetone bath) and Mesyl chloride (0.14 ml, 1.8 mmol) was added dropwise. The reaction mixture was stirred for 1 h at the same temperature. By TLC no starting material (Hex:AcOEt, 3:7), and a less polar compound was formed. Reaction mixture was poured into an aqueous solution of $NaHCO_3$ (20 mL) and product extracted with DCM (20mL, 2x), the organic extracts were combined, dried (Na_2SO_4), and evaporated under vacuum. The crude product (mesylate) was used in the next step without any further purification (>95% pure by NMR); ^1H NMR (400 MHz, Chloroform-d) δ 4.26 (t, J = 6.5 Hz, 2H), 3.57 (t, J = 6.6 Hz, 2H), 3.44 (td, J = 6.2, 2.0 Hz, 4H), 3.01 (s, 3H), 1.92 – 1.78 (m, 4H), 1.76 – 1.62 (m, 4H). Crude mixture from previous step was diluted in Acetonitrile (5 ml) and NaI (247.32 mg, 1.65 mmol) was added, the reaction mixture was stirred at room temperature for 72 h. The reaction mixture was poured into an aqueous solution of $Na_2S_2O_3$ (10%, 20 mL) and product was extracted with DCM (2x20 mL). Organic extracts were combined, dried (NA_2SO_4) and evaporated under vacuum. Crude product was purified by flash chromatography (SiO_2-40g, Grad. Hex:AcOEt, 2 to 20% in 10 min), to give 310 mg of product as an oil (71% yield). ^1H NMR (500 MHz, Chloroform-d) δ 3.57 (t, J = 6.6 Hz, 2H), 3.43 (td, J = 6.3, 2.4 Hz, 4H), 3.22 (t, J = 7.0 Hz, 2H), 2.01 – 1.80 (m, 4H), 1.77 – 1.61 (m, 4H). ^{13}C NMR (151 MHz, cdcl3) δ 70.13, 69.77, 45.13, 30.74, 30.56, 29.68, 27.22, 7.02. LC-MS

(ESI); m/z: $[M+H]^+$ Calcd. for $C_8H_{17}ClO$, 291.001 . Found 291.0060.

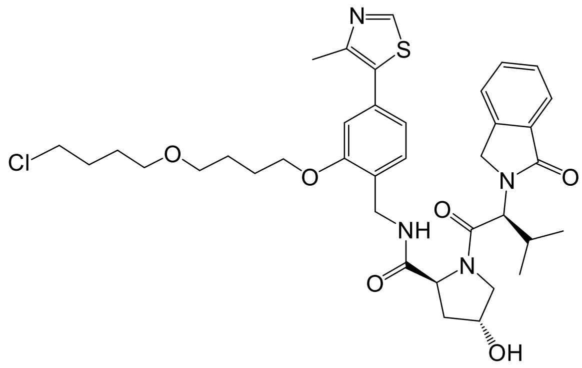

(2S,4R)-N-(2-(4-(4-chlorobutoxy)butoxy)-4-(4-methylthiazol-5-yl)benzyl)-4-hydroxy-1-((S)-3-methyl-2-(1-oxoisoindolin-2-yl)butanoyl)pyrrolidine-2-carboxamide (3). To a mixture of (2S,4R)-4-hydroxy-N-[[2-hydroxy-4-(4-methylthiazol-5-yl)phenyl]methyl]-1-[(2S)-3-methyl-2-(1-oxoisoindo -lin-2-yl)butanoyl]pyrrolidine-2-carboxamide (22 mg, 0.04 mmol) **(2)**[1] and 1-chloro-4-(4-iodobutoxy)butane (13.98 mg, 0.05 mmol) **(1)** in N,N-Dimethylformamide (1 mL) was added Cs_2CO_3 (26.13 mg, 0.08 mmol). After stirring at room temperature for 2 hrs, the reaction mixture was diluted with AcOEt (10 mL) and washed with water (5x10 mL), organic phase was dried (Na_2SO_4, and evaporated under vacuum. Crude product was purified by PTLC (DCM:MeOH:NH_4OH, 90:9:1) to give 25 mg of product (88% yield). ^1H NMR (500 MHz, DMSO-d6) δ 8.99 (s, 1H), 8.36 (t, J = 5.7 Hz, 1H), 7.71 (d, J = 7.5 Hz, 1H), 7.65 – 7.57 (m, 2H), 7.50 (t, J = 7.7 Hz, 1H), 7.33 (d, J = 7.6 Hz, 1H), 7.02 – 6.96 (m, 2H), 5.08 (d, J = 3.9 Hz, 1H), 4.71 (d, J = 10.8 Hz, 1H), 4.59 – 4.18 (m, 6H), 4.07 (t, J = 5.0 Hz, 2H), 3.77 (dd, J = 10.5, 4.2 Hz, 1H), 3.69 (d, J = 10.4 Hz, 1H), 3.63 (t, J = 6.6 Hz, 2H), 3.49 – 3.36 (m, 4H), 2.47 (s, 3H), 2.37 – 2.29 (m, 1H), 2.07 – 2.01 (m, 1H), 1.96 – 1.89 (m, 1H), 1.85 – 1.56 (m, 8H), 0.96 (d, J = 6.4 Hz, 3H), 0.74 (d, J = 6.6 Hz, 3H). ^{13}C NMR (151 MHz, dmso) δ 171.54, 168.09, 167.48, 155.90, 151.48, 147.89, 142.21, 131.60, 131.37, 131.32, 130.98, 127.92, 127.69, 126.96, 123.63, 123.02, 120.77, 111.69, 69.68, 69.14, 68.62, 67.56, 58.70, 57.79, 55.42, 46.82, 45.36, 38.10, 37.06, 29.18, 28.40, 26.63, 25.90, 25.69, 18.89, 18.64, 16.03. LC-MS (ESI); m/z: $[M+H]^+$ Calcd. for $C_{37}H_{48}ClN_4O_6S$, 711.2983 . Found 711.3224.

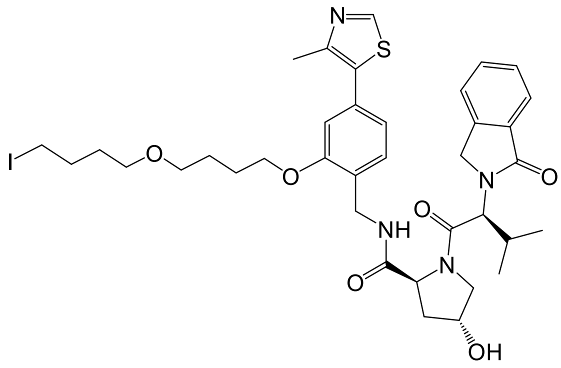

(2S,4R)-4-hydroxy-N-[[2-[4-(4-iodobutoxy)butoxy]-4-(4-methylthiazol-5-yl)phenyl]methyl]-1-[(2S)-3-methyl-2-(1-oxoisoindolin-2-yl)butanoyl]pyrrolidine-2-carboxamide (4). To a

solution of (2S,4R)-N-[[2-[4-(4-chlorobutoxy)butoxy]-4-(4-methylthiazol-5-yl)phenyl]methyl]-4-hydroxy- 1-[(2S)-3-methyl-2-(1-oxoisoindolin-2-yl)butanoyl]pyrrolidine-2-carboxamide **(3)** (23 mg, 0.03 mmol) in Acetone (5 ml) was added NaI (48.47 mg, 0.32 mmol). The reaction mixture was stirred at reflux temperature for 24 h, then the solvent was removed under vacuum and crude product was dissolved in EtOAc (15 mL) and an aqueous solution of Na_2SO_3 (10%, 10 mL), organic layer was separated, washed with water (10 mL), dried (Na_2SO_4) and evaporated under vacuum. Crude product was pure by NMR (>95% purity, 22 mg, 84% yield) no further purification. ^{1}H (400 MHz, Chloroform-d) δ 8.68 (s, 1H), 7.78 (d, J = 7.6 Hz, 1H), 7.52 (t, J = 7.4 Hz, 1H), 7.47 – 7.37 (m, 2H), 7.34 – 7.23 (m, 2H), 6.96 (d, J = 8.7 Hz, 1H), 6.87 (s, 1H), 4.76 (s, 1H), 4.73 (d, J = 6.7 Hz, 1H), 4.64 (t, J = 7.8 Hz, 1H), 4.57 – 4.36 (m, 5H), 4.05 (t, J = 6.2 Hz, 2H), 3.65 (dd, J = 11.5, 3.5 Hz, 1H), 3.49 (t, J = 6.2 Hz, 2H), 3.44 (t, J = 6.2 Hz, 2H), 3.19 (t, J = 6.9 Hz, 2H), 2.58 – 2.45 (m, 1H), 2.53 (s, 3H), 2.47 – 2.27 (m, 2H), 2.12 – 2.00 (m, 2H), 1.98 – 1.84 (m, 3H), 1.79 (dt, J = 9.4, 6.5 Hz, 2H), 1.71 – 1.58 (m, 2H), 0.89 (dd, J = 6.0 Hz, 6H).^{13}C NMR (126 MHz, cdcl3) δ 170.54, 170.47, 169.71, 156.94, 150.41, 148.61, 142.23, 132.35, 131.99, 131.92, 131.73, 129.41, 128.15, 126.47, 123.97, 122.99, 121.65, 112.15, 70.62, 70.13, 69.83, 68.04, 58.81, 58.55, 56.07, 47.59, 39.05, 35.87, 30.75, 30.58, 29.85, 28.89, 26.55, 26.34, 19.23, 16.28, 6.98. LC-MS (ESI); m/z $[M+H]^+$: Calcd. for $C_{37}H_{48}IN_4O_6S$, 803.2339. Found 803.2675.

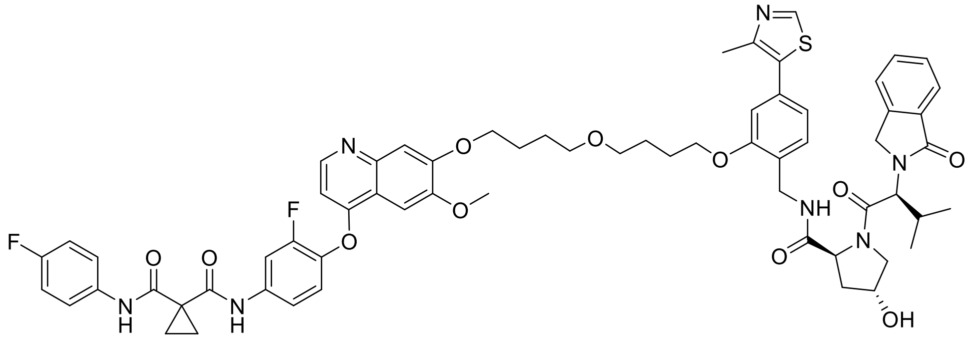

N-(3-fluoro-4-((7-(4-(4-(2-(((2S,4R)-4-hydroxy-1-((S)-3-methyl-2-(1-oxoisoindolin-2-yl)butano -yl)pyrrolidine-2-carboxamido)methyl)-5-(4-methylthiazol-5-yl)phenoxy)butoxy)butoxy)-6-methoxyquinolin-4-yl)oxy)phenyl)-N-(4-fluorophenyl)cyclopropane-1,1-dicarboxamide (PROTAC SJF-6677). To a mixture of compound **(4)** (7.5 mg, 0.01 mmol) and N1'-[3-fluoro-4-[(7-hydroxy-6-methoxy-4-quinolyl)oxy]phenyl] -N1-(4-fluorophenyl)cyclopropane-1,1-dicarboxamide **(5)**[2] (4.72 mg, 0.01 mmol) in N,N-Dimethylformamide (1 mL) was added Cs_2CO_3 (6.09 mg, 0.02 mmol). After stirring at room temperature for 12 hrs (overnight), the reaction mixture was diluted with AcOEt (10 mL) and washed with brine (5x10 mL), organic phase was dried (Na_2SO_4, and evaporated under vacuum. Crude product was purified by PTLC (DCM:MeOH:NH_4OH, 90:9:1) to give 5.7 mg of product (51% yield). ^{1}H NMR (400 MHz, DMSO-d6) δ 10.38 (s, 1H), 10.01 (s, 1H), 8.97 (s, 1H), 8.45 (d, J = 5.2 Hz, 1H), 8.37 (t, J = 6.0 Hz, 1H), 7.90 (dd, J = 13.2, 2.4 Hz, 1H), 7.71 (d, J = 7.6 Hz, 1H), 7.65 – 7.38 (m, 8H), 7.34 (d, J = 7.6 Hz, 1H), 7.15 (t, J = 8.9 Hz, 2H), 7.04 – 6.87 (m, 2H), 6.41 (d, J = 5.2 Hz, 1H), 5.09 (d, J = 3.9 Hz, 1H), 4.71 (d, J = 10.8 Hz, 1H), 4.50 (dd, 2H), 4.44 – 4.19 (m, 4H), 4.16 (t, J = 6.3 Hz, 2H), 4.07 (t, J = 6.0 Hz, 2H), 3.77 (dd, J = 10.5, 4.2 Hz, 1H), 3.69 (d, J = 10.5 Hz, 1H), 3.47 (t, J = 6.3 Hz, 4H), 2.46 (s, 3H), 2.37 – 2.28 (m, 1H), 2.09 – 2.00 (m, 1H), 1.96 – 1.63 (m, 8H), 1.58 – 1.38 (m, 4H), 0.96 (d, J = 6.4 Hz, 3H), 0.72 (d, J = 6.6 Hz, 3H). ^{13}C NMR (151 MHz, DMSO-d6) δ 171.53 , 168.29 , 168.09 , 167.90 , 167.47 , 159.35 ,

158.29 (d, J = 240.3 Hz), 155.91 , 153.26 (d, J = 245.1 Hz), 151.98 , 151.43 , 149.59 , 148.74 , 147.88 , 146.31 , 142.20 , 138.02 (d, J = 9.9 Hz), 135.65 (d, J = 12.3 Hz), 135.20 (d, J = 2.4 Hz), 131.58 , 131.34 (d, J = 8.6 Hz), 130.98 , 127.90 , 127.70 , 126.98 , 123.82 , 123.61 , 123.01 , 122.45 (d, J = 7.7 Hz), 120.76 , 116.92 , 115.04 (d, J = 22.2 Hz), 114.42 , 111.69 , 108.97 (d, J = 23.0 Hz), 108.39 , 101.91 , 98.98 , 69.70 , 69.63 , 68.63 , 68.18 , 67.57 , 58.70 , 57.78 , 55.78 , 55.43 , 46.81 , 38.11 , 37.06 , 31.93 , 28.40 , 25.97 , 25.73 , 25.48 , 18.88 , 18.62 , 16.02 , 15.34. LC-MS (ESI); m/z: $[M+H]^+$ Calcd. for $C_{64}H_{68}F_2N_7O_{11}S$, 1180.4665. Found 1180.4974.

Scheme 2.- Synthesis of PROTAC **SJF-6683**

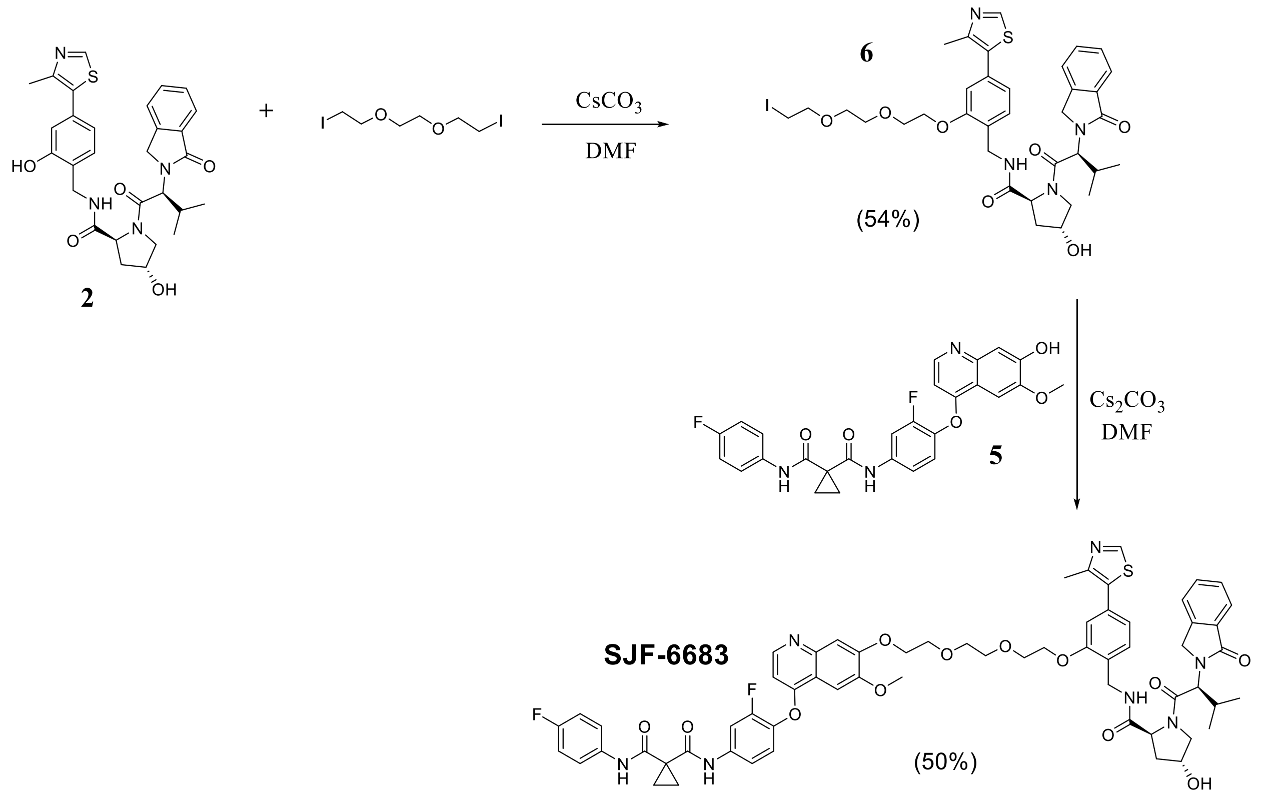

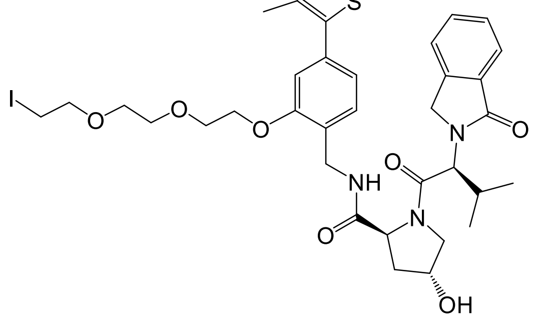

(2S,4R)-4-hydroxy-N-(2-(2-(2-(2-iodoethoxy)ethoxy)ethoxy)-4-(4-methylthiazol-5-yl)benzyl)-1-((S)-3-methyl-2-(1-oxoisoindolin-2-yl)butanoyl)pyrrolidine-2-carboxamide (6).
To a mixture of (2S,4R)-4-hydroxy-N-[[2-hydroxy-4-(4-methylthiazol-5-yl)phenyl]methyl] -1-[(2S)-3-methyl-2-(1-oxoisoindolin-2-yl)butanoyl]pyrrolidine-2-carboxamide **(2)**[1] (37 mg, 0.07 mmol) and 1,2-bis(2-iodoethoxy)ethane (324.35 mg, 0.88 mmol) in N,N-Dimethylformamide (1 mL) was added Cs_2CO_3 (142.82 mg, 0.44 mmol). After stirring at room temperature for 2 hrs, the reaction mixture was diluted with AcOEt (10 mL) and washed with water (5x10 mL), organic phase was dried (Na_2SO_4), and evaporated under vacuum. Crude product was filtered over a short column of SiO_2 (gradient, DCM 100% to DCM:MeOH:NH_4OH, 90:9:1) to remove the excess of the bis-iodo reactant, then crude product was purified by PTLC (DCM:MeOH:NH_4OH, 90:9:1) to give 29 mg of product (54% yield). ^1H NMR (400 MHz, DMSO-d6) δ 8.98 (s, 1H), 8.35 (t, J = 5.7 Hz, 1H), 7.71 (d, J = 7.5 Hz, 1H), 7.66 – 7.56 (m, 2H), 7.49 (t, J = 7.3 Hz, 1H), 7.34 (d, J = 7.8 Hz, 1H), 7.05 (bs, 1H), 7.01 (d, J = 7.8 Hz, 1H), 5.09 (bs, 1H), 4.72 (d, J = 10.8 Hz, 1H), 4.64 – 4.02 (m, 8H), 3.91 – 3.49 (m, 10H), 3.29 (t, J = 6.2 Hz, 2H), 2.47 (s, 3H), 2.40 – 2.24 (m, 1H), 2.10 – 1.98 (m, 1H), 1.99 – 1.85 (m, 1H), 0.97 (d, J = 6.4 Hz, 3H), 0.74 (d, J = 6.5 Hz, 3H). ^{13}C NMR (151 MHz, dmso) δ 171.59, 168.12, 167.52, 155.88, 151.50, 147.95, 142.21, 131.61, 131.38, 131.29, 130.99, 127.93, 127.70, 127.22, 123.64, 123.04, 121.11, 112.19, 71.01, 70.12, 69.41, 69.05, 68.66, 67.96, 58.75, 57.82, 55.46, 46.86, 38.14, 37.16, 28.43, 18.93, 18.66, 16.08, 5.46. LC-MS (ESI); m/z: $[M+H]^+$ Calcd. for $C_{35}H_{44}IN_4O_7S$, 791.1975. Found 791.2036.

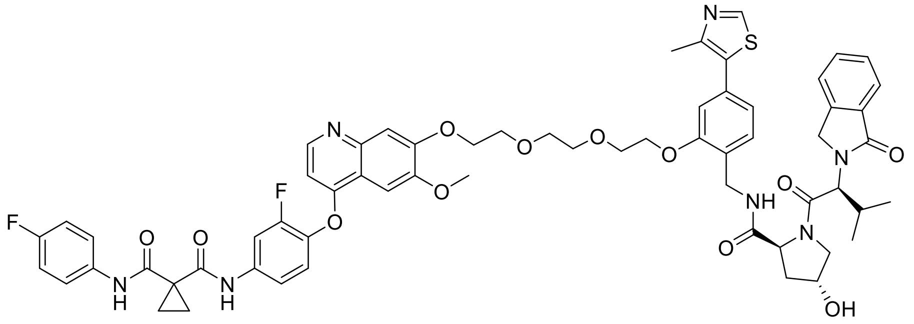

N-(3-fluoro-4-((7-(2-(2-(2-(2-(((2S,4R)-4-hydroxy-1-((S)-3-methyl-2-(1-oxoisoindolin-2-yl)-butanoyl)pyrrolidine-2-carboxamido)methyl)-5-(4-methylthiazol-5-yl)phenoxy)ethoxy)ethoxy) -ethoxy)-6-methoxyquinolin-4-yl)oxy)phenyl)-N-(4-fluorophenyl)cyclopropane-1,1-dicarboxamide (PROTAC SJF-6683).
To a mixture of compound **(6)** and N1'-[3-fluoro-4-[(7-hydroxy-6-methoxy-4-quinolyl)oxy]phenyl] -N1-(4-fluorophenyl)cyclopropane-1,1-dicarboxamide **(5)**[2] (4.72 mg, 0.01 mmol) in N,N-Dimethylformamide (1 mL) was added Cs_2CO_3 (6.09 mg, 0.02 mmol). After stirring at room temperature for 12 hrs (overnight), the reaction mixture was diluted with AcOEt (10 mL) and washed with brine (5x10 mL), organic phase was dried (Na_2SO_4), and evaporated under vacuum. Crude product was purified by PTLC (DCM:MeOH, 9:1 2X) to give 9.7 mg of product (50% yield). ^1H NMR (400 MHz, DMSO-d6) δ 10.39 (bs, 1H), 10.01 (bs, 1H), 8.97 (s, 1H), 8.46 (d, J = 4.8 Hz, 1H), 8.37 (bs, 1H), 7.90 (d, J = 12.9 Hz, 1H), 7.81 – 7.26 (m, 10H), 7.15 (t, J = 8.7 Hz, 2H), 7.10 – 6.94 (m, 2H), 6.41 (d, J = 4.7 Hz, 1H), 5.09 (d, 1H), 4.71 (d, J = 10.7 Hz, 1H), 4.59 – 4.11 (m, 10H), 3.94 (s, 3H), 3.90 – 3.45 (m, 10H), 2.46 (s, 3H), 2.38 – 2.19 (m, 1H), 2.13 – 1.99 (m, 1H), 1.98 – 1.84 (m, 1H), 1.69 – 1.30 (m, 4H), 0.95 (d, J = 6.0 Hz, 2H), 0.71 (d, J = 6.2 Hz, 2H). ^{13}C NMR (151 MHz, DMSO-d6) δ 171.56 , 168.30 , 168.09 , 167.91 , 167.47 , 159.30 ,

158.29 (d, J = 239.9 Hz), 155.87 , 153.27 (d, J = 245.5 Hz), 151.81 , 151.45 , 149.47 , 148.82 , 147.93 , 146.33 , 142.19 , 138.42 – 137.45 (m), 135.64 (d, J = 11.8 Hz), 135.21 , 131.57 , 131.37 , 131.26 , 130.98 , 127.90 , 127.66 , 127.20 , 123.82 , 123.59 , 123.01 , 122.44 (d, J = 7.9 Hz), 121.08 , 117.44 – 116.65 (m), 115.05 (d, J = 22.1 Hz), 114.55 , 112.16 , 108.98 (d, J = 22.3 Hz), 108.64 , 101.96 , 99.03. LC-MS (ESI); m/z: $[M+H]^+$ Calcd. for $C_{62}H_{64}F_2N_7O_{12}$, 1168.4301. Found 1168.4722.

Scheme 3.- Synthesis of PROTAC **SJF-6685**

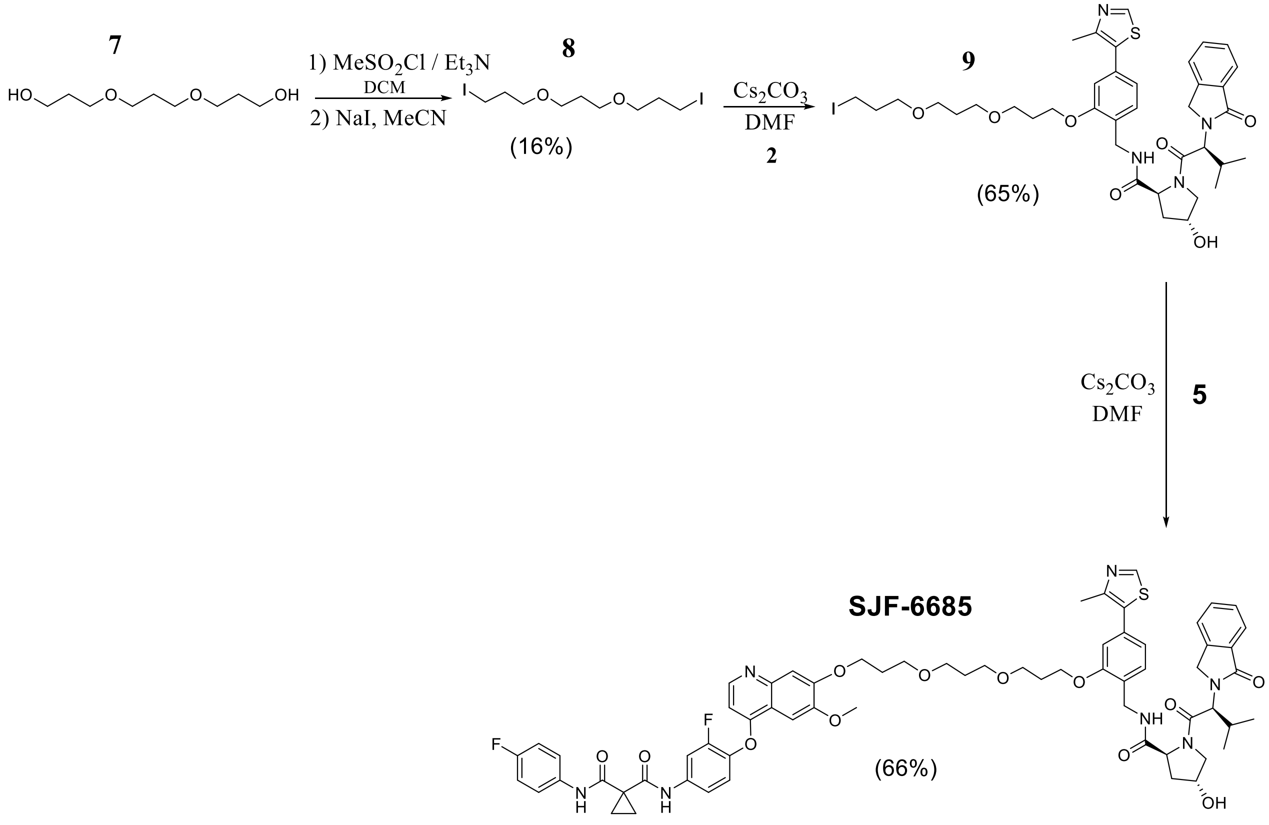

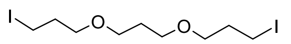

1,3-bis(3-iodopropoxy)propane (8). To a solution of 3-[3-(3-hydroxypropoxy)propoxy]propan-1-ol (334 mg, 1.74 mmol) in Dichloromethane (30 ml) was added TEA (0.73 ml, 5.21 mmol), then reaction mixture was cooled to -10 ºC (water ice/acetone bath) and Mesyl chloride (0.15 ml, 1.91 mmol) was added dropwise. The reaction mixture was stirred for 1 h at the same temperature. By TLC no starting material (Hex:AcOEt, 3:7), and a less polar compound was formed. Reaction mixture was poured into an aqueous solution of $NaHCO_3$ (5 mL) and product extracted with DCM (5 mL, 2x), the organic extracts were combined, dried (Na_2SO_4), and evaporated under vacuum. The crude product (469 mg, quantitative yield) was used in the next step. The crude product from above (mixture of mesylates) was used without any further purification. Thus, 3-[3-(3-hydroxypropoxy)propoxy]propyl methanesulfonate (469 mg, 1.73 mmol) was diluted in Acetonitrile (100 ml) and NaI (1300.22 mg, 8.67 mmol) was added, the reaction mixture was stirred at room temperature for 12 h (overnight) and then at reflux temperature for 4 h. The reaction was filtered through a Celite pad under vacuum, and the filtrate was evaporated to dryness. The residue was dissolved in AcOEt (50 mL) and water (50 mL), the organic layer was separated, dried (Na_2SO_4) and evaporated under vacuum. Crude product was purified by flash chromatography (SiO_2-40g, grad. Hex:AcOEt, 2 to 60% AcOEt in 20 min), to give 114 mg of product as an oil (16% yield) of a less polar product **(8)** (bis-iodide) and 155 mg (30% yield) of a more polar product (mono-iodide): ^{1}H NMR (400 MHz, Chloroform-d) δ 3.49 (dtd, J = 12.7, 6.0, 1.1 Hz, 8H), 3.28 (t, J = 7.1 Hz, 4H), 2.05 (p, J = 6.7 Hz, 4H), 1.95 – 1.67 (m, 2H). ^{13}C NMR (101 MHz, cdcl3) δ 70.21, 67.96, 33.58, 30.17, 3.70. HRMS (ESI); m/z [M+H]$^+$: Calcd. for $C_9H_{19}I_2O_2$, 412.9474. Found 412.9474. More polar product(mono-iodide): ^{1}H NMR (400 MHz, Chloroform-d) δ 3.77 (q, J = 4.8 Hz, 1H), 3.62 (t, J = 5.7 Hz, 2H), 3.51 (q, J = 6.7 Hz, 4H), 3.47 (t, J = 5.9 Hz, 2H), 3.27 (t, J = 6.8 Hz, 2H), 2.43 (bs, 1H), 2.04 (p, J = 6.3 Hz, 2H), 1.93 – 1.72 (m, 4H). ^{13}C NMR (101 MHz, cdcl3) δ 70.62, 70.23, 68.42, 68.04, 62.44, 33.49, 32.11, 30.12, 3.67. LC-MS (ESI); m/z [M+H]$^+$: Calcd. for $C_9H_{20}IO_3$.

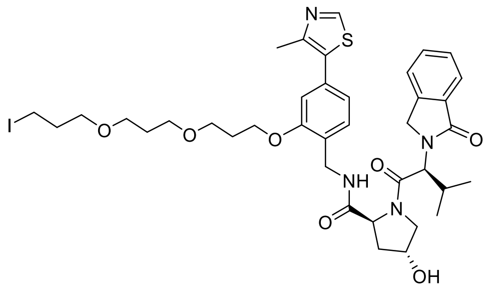

(2S,4R)-4-hydroxy-N-(2-(3-(3-(3-iodopropoxy)propoxy)propoxy)-4-(4-methylthiazol-5-yl)benzyl)-1-((S)-3-methyl-2-(1-oxoisoindolin-2-yl)butanoyl)pyrrolidine-2-carboxamide (9). To a mixture of **(2)**[1] (30 mg, 0.05 mmol) and 1,3-bis(3-iodopropoxy)propane **(8)** (112.65 mg, 0.27 mmol) in N,N-Dimethylforma- mide (1 mL) was added Cs_2CO_3 (53.45 mg, 0.16 mmol). After stirring at room temperature for 4 hrs, the reaction mixture was diluted with AcOEt (10 mL) and washed with water (5x10 mL), organic phase was dried (Na_2SO_4, and evaporated under vacuum. Crude product was purified over a short column of SiO_2 (Gradient, DCM 100% to DCM:MeOH:NH_4OH, 90:9:1) to give 30 mg of product (65% yield). ^{1}H NMR (400 MHz, DMSO-d6) δ 8.99 (s, 1H), 8.38 (t, J = 5.9 Hz, 1H), 7.71 (d, J = 7.5 Hz, 1H), 7.68 – 7.55 (m, 2H), 7.55 –

7.44 (m, 1H), 7.34 (d, J = 7.6 Hz, 1H), 7.12 – 6.91 (m, 2H), 5.10 (d, J = 4.1 Hz, 1H), 4.71 (d, J = 10.9 Hz, 1H), 4.53 (dd, 2H), 4.44 – 4.17 (m, 4H), 4.11 (t, J = 6.1 Hz, 2H), 3.78 (dd, J = 10.5, 4.3 Hz, 1H), 3.69 (d, J = 10.7 Hz, 1H), 3.56 (t, J = 6.2 Hz, 2H), 3.44 (t, J = 6.3 Hz, 2H), 3.40 (t, J = 6.4 Hz, 2H), 3.34 (t, 2H), 3.24 (t, J = 6.7 Hz, 2H), 2.47 (s, 3H), 2.38 – 2.25 (m, 1H), 2.22 – 1.82 (m, 6H), 1.72 (p, J = 6.3 Hz, 2H), 0.97 (d, J = 6.5 Hz, 3H), 0.74 (d, J = 6.6 Hz, 3H). ^{13}C 13C NMR (151 MHz, dmso) δ 171.49, 168.08, 167.46, 155.87, 151.46, 147.88, 142.19, 131.57, 131.37, 131.28, 131.00, 127.90, 127.79, 127.00, 123.61, 123.01, 120.84, 111.66, 69.41, 68.61, 67.06, 67.02, 66.58, 64.82, 58.68, 57.77, 55.42, 46.81, 38.09, 37.06, 32.83, 29.57, 29.10, 28.39, 18.89, 18.63, 16.04, 16.04. LC-MS (ESI); m/z: $[M+H]^+$ Calcd. for $C_{38}H_{50}IN_4O_7S$, 833.2444. Found 833.2540.

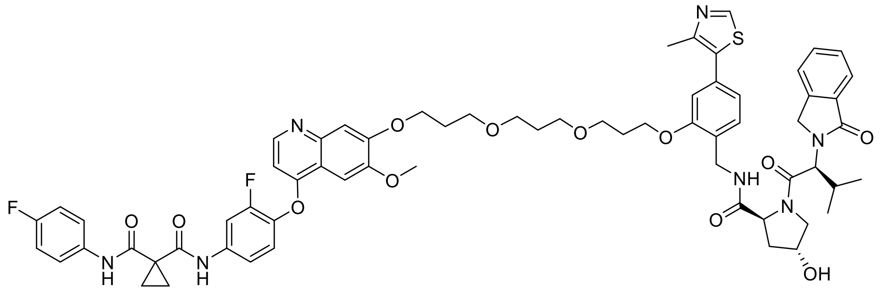

N-(3-Fluoro-4-((7-(3-(3-(3-(2-(((2S,4R)-4-hydroxy-1-((S)-3-methyl-2-(1-oxoisoindolin-2-yl)butano-yl)pyrrolidine-2-carboxamido)methyl)-5-(4-methylthiazol-5-yl)phenoxy)propoxy)-propoxy)propoxy)-6-methoxyquinolin-4-yl)oxy)phenyl)-N-(4-fluorophenyl)cyclopropane-1,1-dicarboxamide (PROTAC SJF-6685). To a mixture of **(9)** (15 mg, 0.02 mmol) and **(2)**[1] (9.1 mg, 0.02 mmol) in N,N-Dimethylformamide (1 mL) was added Cs_2CO_3 (11.74 mg, 0.04 mmol). After stirring at room temperature for 12 hrs (overnight), the reaction mixture was diluted with AcOEt (10 mL) and washed with brine (5x10 mL), organic phase was dried (Na_2SO_4, and evaporated under vacuum. Crude product was purified by PTLC (DCM:MeOH, 9:1) to give 14.5 mg of product (66% yield). ^{1}H NMR (400 MHz, DMSO-d6) δ 10.40 (s, 1H), 10.02 (s, 1H), 8.97 (s, 1H), 8.45 (d, J = 5.2 Hz, 1H), 8.39 (t, J = 5.5 Hz, 1H), 7.90 (d, J = 13.2 Hz, 1H), 7.78 – 7.28 (m, 10H), 7.16 (t, J = 8.6 Hz, 2H), 7.05 – 6.89 (m, 2H), 6.40 (d, J = 5.1 Hz, 1H), 5.11 (d, J = 3.9 Hz, 1H), 4.71 (d, J = 10.8 Hz, 1H), 4.64 – 4.21 (m, 6H), 4.18 (t, J = 6.0 Hz, 2H), 4.08 (t, J = 5.8 Hz, 2H), 3.93 (s, 3H), 3.82 – 3.66 (m, 2H), 3.48 (dt, J = 33.6, 5.4 Hz, 8H), 2.46 (s, 3H), 2.31 (dd, J = 17.1, 6.3 Hz, 1H), 2.18 – 1.81 (m, 6H), 1.74 (p, J = 6.0 Hz, 2H), 1.63 – 1.34 (m, 4H), 0.96 (d, J = 6.3 Hz, 3H), 0.72 (d, J = 6.4 Hz, 3H). ^{13}C NMR (151 MHz, DMSO-d6) δ 171.51 , 168.29 , 168.09 , 167.91 , 167.47 , 159.30 , 158.29 (d, J = 240.2 Hz), 155.88 , 153.26 (d, J = 245.2 Hz), 151.91 , 151.42 , 149.55 , 148.80 , 147.87 , 146.36 , 142.19 , 138.01 (d, J = 9.8 Hz), 135.66 (d, J = 12.3 Hz), 135.20 (d, J = 2.5 Hz), 131.57 , 131.37 , 131.28 , 131.01 , 127.85 (d, J = 15.1 Hz), 127.01 , 123.81 , 123.60 , 123.01 , 122.45 (d, J = 7.9 Hz), 120.84 , 116.91 , 115.11 , 114.97 , 114.47 , 111.64 , 108.97 (d, J = 22.9 Hz), 108.48 , 101.93 , 99.01 , 68.63 , 67.09 , 67.08 , 66.56 , 66.55 , 65.42 , 64.79 , 58.70 , 57.78 , 55.77 , 55.44 , 46.81 , 38.10 , 37.06 , 31.91 , 29.61 , 29.06 , 28.89 , 28.40 , 18.87 , 18.62 , 16.00 , 15.35. LC-MS (ESI); m/z: $[M+H]^+$ Calcd. for $C_{65}H_{70}F_2N_7O_{12}S$, 1210.4771. Found 1210.5192.

Scheme 4.- Synthesis of PROTAC **SJF-6690**

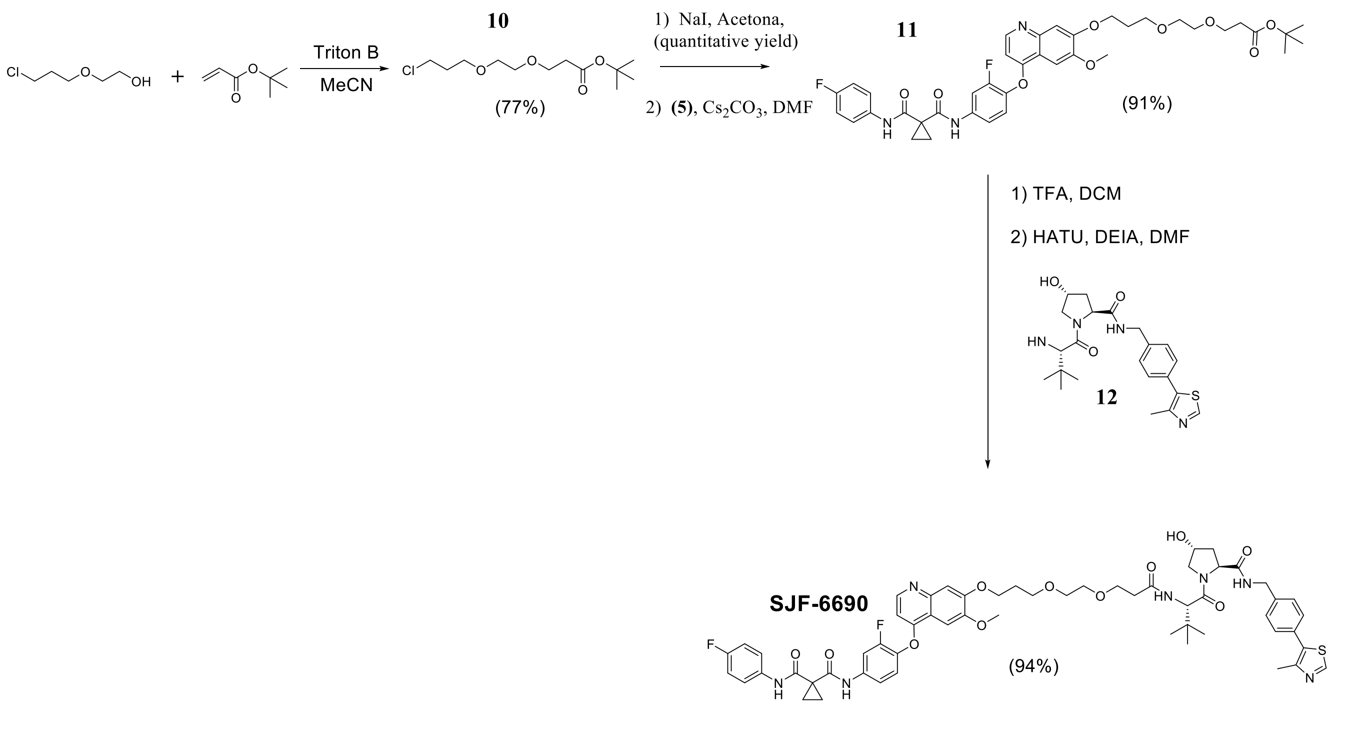

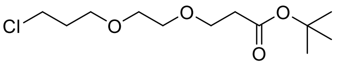

tert-butyl 3-(2-(3-chloropropoxy)ethoxy)propanoate (10). To a solution of 2-(3-chloropropoxy)ethan-1-ol (1.48 g, 10.68 mmol) in acetonitrile (20 mL) was added tert-butyl prop-2-enoate (7.75 ml, 128.17 mmol) followed by Triton B (447 mg, 1.06 mmol, in 40% by weight in water). The mixture was stirred at room temperature for 12 hours (overnight). The mixture was concentrated in vacuum and crude product was purified by CC (SiO_2-80g, gradient Hex:AcOEt, 98:2 to 8:2) to give 2.21 g of product as an oil (77% yield). ^{1}H NMR (500 MHz, Chloroform-d) δ 3.71 (t, J = 6.6 Hz, 2H), 3.63 (t, J = 6.4 Hz, 2H), 3.62 – 3.54 (m, 8H), 2.50 (t, J = 6.6 Hz, 2H), 2.02 (p, J = 6.2 Hz, 2H), 1.44 (s, 9H). ^{13}C NMR (126 MHz, cdcl3) δ 171.02, 80.66, 77.36, 70.46, 67.79, 67.08, 42.09, 36.44, 32.86, 28.25. LC-MS (ESI); m/z: $[M+H]^+$ Calcd. for $C_{12}H_{23}ClO_4Na$, 289.1182. Found 289.1364.

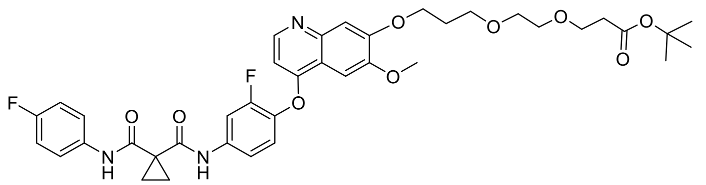

tert-Butyl 3-(2-(3-((4-(2-fluoro-4-(1-((4-fluorophenyl)carbamoyl)cyclopropane-1-carboxamido) -phenoxy)-6-methoxyquinolin-7-yl)oxy)propoxy)ethoxy)propanoate (11). To a solution of **(10)** (188 mg, 0.70 mmol) in Acetone (15 ml) was added NaI (528.19 mg, 3.52 mmol). The reaction mixture was stirred at reflux temperature for 24 h, then the solvent was removed under vacuum and crude product was dissolved in EtOAc (15 mL) and an aqueous solution of Na_2SO_3 (10%, 10 mL), organic layer was separated, washed with water (10 mL), dried (Na_2SO_4) and evaporated under vacuum. Crude product was pure by NMR (>95% purity, 245 mg, 97% yield), it was used in the next step without any further purification; ^{1}H NMR (500 MHz, Chloroform-d) δ 3.71 (t, J = 6.6 Hz, 2H), 3.64 – 3.55 (m, 4H), 3.52 (t, J = 5.9 Hz, 2H), 3.27 (t, J = 6.8 Hz, 2H), 2.50 (t, J = 6.6 Hz, 2H), 2.05 (p, J = 6.3 Hz, 2H), 1.44 (s, 9H).^{13}C 13C NMR (126 MHz, cdcl3) δ 171.01, 80.66, 70.68, 70.45, 70.44, 67.06, 36.42, 33.54, 28.25, 3.57. To a mixture of **(5)**[2] (13.3 mg, 0.03 mmol) and tert-butyl 3-[2-(3-iodopropoxy)ethoxy]propanoate from above (18.85 mg, 0.05 mmol) in N,N-Dimethylformamide (1 mL) was added Cs_2CO_3 (17.15 mg, 0.05 mmol). After stirring at room temperature for 12 hrs (overnight), the reaction mixture was diluted with AcOEt (10 mL) and washed with water (5x5 mL), organic phase was evaporated under vacuum. Crude product was purified by PTLC (DCM:MeOH, 9:1) to give 17 mg of product (91% yield). ^{1}H NMR (500 MHz, DMSO-d6) δ 10.39 (s, 1H), 10.01 (s, 1H), 8.45 (d, J = 5.0 Hz, 1H), 7.89 (d, J = 13.1 Hz, 1H), 7.73 – 7.58 (m, 2H), 7.55 – 7.46 (m, 2H), 7.46 – 7.31 (m, 2H), 7.14 (t, J = 8.7 Hz, 2H), 6.40 (d, J = 4.3 Hz, 1H), 4.19 (t, J = 4.9 Hz, 2H), 3.94 (s, 3H), 3.63 – 3.52 (m, 4H), 3.53 – 3.42 (m, 4H), 2.38 (t, J = 6.1 Hz, 2H), 2.10 – 1.94 (m, 2H), 1.56 – 1.40 (m, 4H), 1.35 (s, 9H). ^{13}C NMR (151 MHz, DMSO-d6) δ 170.80 , 168.70 , 168.32 , 159.72 , 158.71 (d, J = 240.1 Hz), 153.68 (d, J = 245.1 Hz), 152.30 , 149.97 , 149.22 , 146.79 , 138.42 (d, J = 9.8 Hz), 136.09 (d, J = 12.4 Hz), 135.60 , 122.86 (d, J = 7.9 Hz), 117.32 (d, J = 3.2 Hz), 115.52 , 115.37 , 114.90 , 109.40 (d, J = 22.8 Hz), 108.93 , 102.36 , 99.43 , 80.08 , 70.07 , 70.00 , 67.28 , 66.68 , 65.84 , 56.19 , 36.26 , 32.30 , 29.35 , 28.13 , 15.77. LC-MS (ESI); m/z: $[M+H]^+$ Calcd. for $C_{39}H_{44}F_2N_3O_9$, 736.3045. Found 736.3098.

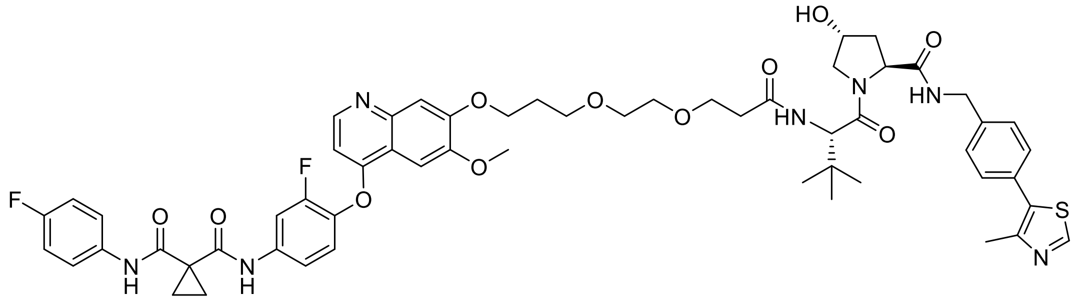

N-(3-Fluoro-4-((7-(3-(2-(3-(((S)-1-((2S,4R)-4-hydroxy-2-((4-(4-methylthiazol-5-yl)benzyl) carbamoyl)pyrrolidin-1-yl)-3,3-dimethyl-1-oxobutan-2-yl)amino)-3-oxopropoxy)ethoxy)-

propoxy)-6-methoxyquinolin-4-yl)oxy)phenyl)-N-(4-fluorophenyl)cyclopropane-1,1-dicarboxa -mide (PRPTAC SJF-6690). A solution of tert-butyl ester **(11)** (10.3 mg, 0.01 mmol) in a mixture of TFA (1 ml) and Dichloromethane (2 ml) was stirred for 1 h. Then the solvent was removed under vacuum and crude product was dried under high vacuum for 2 h. Crude product was used in the next step without any further purification (9.5 mg, quantitative yield). LC-MS (ESI); m/z: $[M+H]^+$ Calcd. for $C_{35}H_{36}F_2N_3O_9$, 680.2419. Found 680.2421.

To a solution of the product from above (8.79 mg, 0.01 mmol) and (2S,4R)-1-[(2S)-2-amino-3,3-dimethyl-butanoyl]-4-hydroxy-N-[[4-(4-methylthiazol-5-yl)phenyl]methyl]-pyrrolidine-2-carboxa -mide;hydrochloride (7.25 mg, 0.02 mmol)) (**12**)[3] in N,N-Dimethylformamide (2 ml) was added N,N-Diisopropylethylamine (0.17 ml, 0.99 mmol) and O-(7-Azabenzotriazol-1-yl)-N,N,N',N'-tetramethyluronium hexafluorophosphate (7.38 mg, 0.02 mmol) at room temperature. The reaction mixture was stirred for 12 h (overnight) at the same temperature. TLC (DCM:MeOH:NH_4OH, 90:9:1) shows no starting materials. Reaction mixture was diluted with ACOEt (10 mL), washed with water (4x10 mL), dried (Na_2SO_4) and evaporated under vacuum. Crude product was purified by PTLC (DCM:MeOH:NH_4OH, 90:9:1), to give 14.5 mg of product (94 % yield). ^1H NMR (500 MHz, DMSO-d6) δ 10.39 (s, 1H), 10.01 (s, 1H), 8.96 (s, 1H), 8.58 (t, J = 5.7 Hz, 1H), 8.47 (d, J = 5.1 Hz, 1H), 7.91 (t, J = 10.0 Hz, 2H), 7.64 (d, J = 13.2 Hz, 2H), 7.52 (d, J = 6.2 Hz, 2H), 7.49 – 7.25 (m, 5H), 7.15 (t, J = 8.5 Hz, 2H), 6.41 (d, J = 5.1 Hz, 1H), 5.14 (d, J = 3.4 Hz, 1H), 4.55 (d, J = 9.3 Hz, 1H), 4.53 – 4.28 (m, 3H), 4.21 (dt, J = 11.3, 5.6 Hz, 4H), 3.94 (s, 3H), 3.78 – 3.41 (m, 9H), 2.61 – 2.50 (m, 1H), 2.43 (s, 3H), 2.35 (dt, J = 13.9, 5.8 Hz, 1H), 2.12 – 1.97 (m, 3H), 1.95 – 1.84 (m, 1H), 1.48 (s, 4H), 0.92 (s, 9H). ^{13}C NMR (151 MHz, DMSO-d6) δ 172.37 , 170.39 , 169.96 , 168.72 , 168.33 , 159.79 , 158.71 (d, J = 240.2 Hz), 153.67 (d, J = 245.3 Hz), 152.34 , 151.84 , 149.99 , 149.17 , 148.12 , 146.67 , 139.90 , 138.43 (d, J = 9.7 Hz), 136.07 (d, J = 12.4 Hz), 135.60 (d, J = 2.6 Hz), 131.58 , 130.05 , 129.05 , 127.83 , 124.21 , 122.87 (d, J = 7.8 Hz), 117.33 , 115.45 (d, J = 22.2 Hz), 114.90 , 109.40 (d, J = 23.0 Hz), 108.84 , 102.37 , 99.45 , 70.01 , 69.92 , 69.31 , 67.39 , 67.33 , 65.87 , 59.14 , 56.81 , 56.72 , 56.21 , 42.08 , 38.37 , 36.10 , 35.78 , 32.32 , 29.32 , 26.73 , 16.36 , 15.77. LC-MS (ESI); m/z $[M+H]^+$: Calcd. for $C_{57}H_{64}F_2N_7O_{11}S$, 1092.4352. Found 1092.4733.

Scheme 5.- Synthesis of PROTAC **SJF-6693**

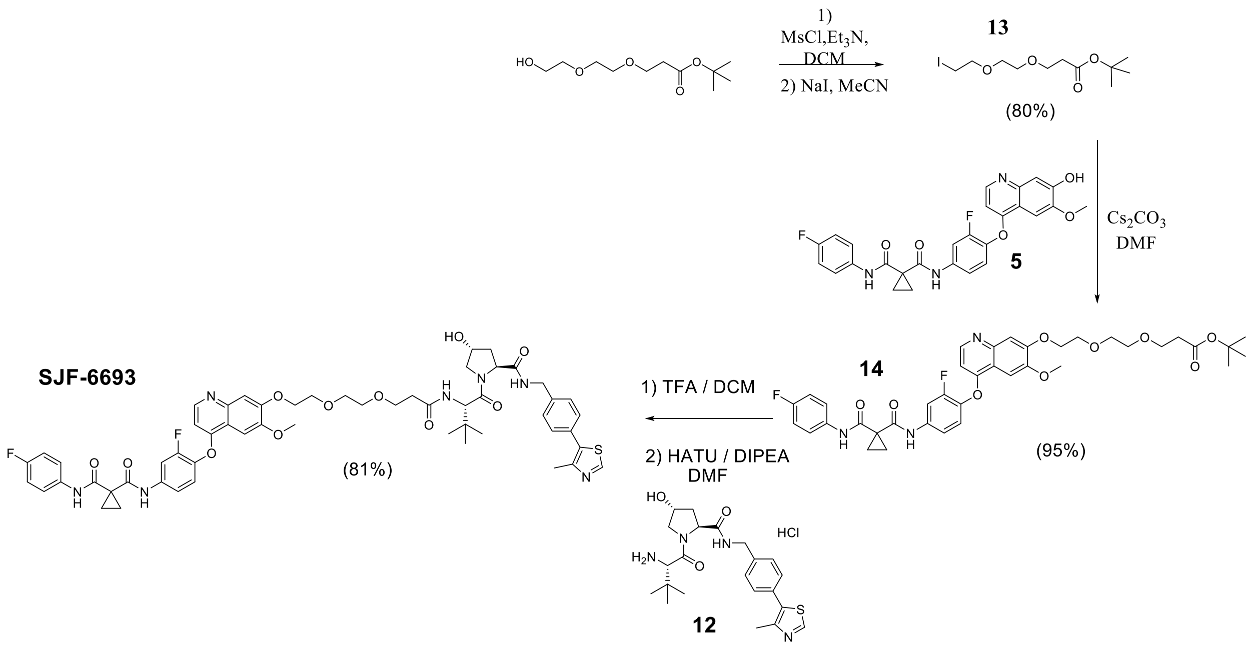

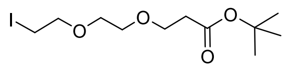

tert-Butyl 3-(2-(2-iodoethoxy)ethoxy)propanoate (13). To a solution of tert-butyl 3-[2-(2-hydroxyethoxy)ethoxy]propanoate (350 mg, 1.49 mmol) in DCM (15 ml) was added TEA (0.62 ml, 4.48 mmol), then the reaction mixture was cooled to 0 °C (water ice/acetone bath) and Mesyl chloride (0.14 ml, 1.79 mmol) was added dropwise. The reaction mixture was stirred for 1 h at room temperature. By TLC no starting material (Hex:AcOEt, 3:7). Reaction mixture was poured into an aqueous solution of $NaHCO_3$ (20 mL) and product extracted with DCM (20mL, 2x), the organic extracts were combined, dried (Na_2SO_4), and evaporated under vacuum. The crude product (mesylate) was used in the next step without any further purification (>95% pure by NMR): ^{1}H NMR (500 MHz, Chloroform-d) δ 4.44 – 4.28 (m, 2H), 3.76 (dd, J = 5.2, 3.8 Hz, 2H), 3.70 (t, J = 6.4 Hz, 2H), 3.68 – 3.57 (m, 4H), 3.07 (s, 3H), 2.49 (t, J = 6.4 Hz, 2H), 1.45 (s, 9H).

Crude mixture from previous step was diluted in Acetonitrile (5 ml) and NaI (335.88 mg, 2.24 mmol) was added, the reaction mixture was stirred at 70 °C for 12 h (overnight). By TLC no starting material (Hex:AcOEt, 7:3), the reaction was poured into an aqueous solution of $Na_2S_2O_3$ (10%, 50 mL) and product was extracted with DCM (2x50 mL). Organic extracts were combined, dried (Na_2SO_4) and evaporated under vacuum. Crude product was purified by flash chromatography (SiO_2-40g, grad. Hex:AcOEt, 2 to 40% in 15 min), to give 413 mg of product as an oil (80% yield). ^{1}H NMR (500 MHz, Chloroform-d) δ 3.73 (dt, J = 13.5, 6.6 Hz, 4H), 3.68 – 3.54 (m, 4H), 3.24 (t, J = 6.9 Hz, 2H), 2.50 (t, J = 6.5 Hz, 2H), 1.44 (s, 9H). ^{13}C NMR (151 MHz,

cdcl3) δ 171.00, 80.69, 72.11, 70.50, 70.27, 67.09, 36.39, 28.25, 3.02. LC-MS (ESI); m/z: $[M+Na]^+$ Calcd. for $C_{11}H_{21}IO_4Na$, 367.0382. Found 367.0943.

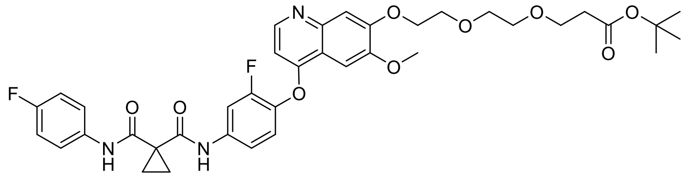

tert-Butyl 3-(2-(2-((4-(2-fluoro-4-(1-((4-fluorophenyl)carbamoyl)cyclopropane-1-carboxamido) -phenoxy)-6-methoxyquinolin-7-yl)oxy)ethoxy)ethoxy)propanoate (14). To a mixture of N1'-[3-fluoro-4-[(7-hydroxy-6-methoxy-4-quinolyl)oxy]phenyl]-N1-(4-fluoro - phenyl)cyclopropane-1,1-dicarboxamide **(5)**[2] (10.3 mg, 0.02 mmol) and and tert-butyl 3-[2-(2-iodoethoxy)ethoxy]propanoate **(13)** (14.03 mg, 0.04 mmol) in N,N-Dimethylformamide (1 mL) was added Cs_2CO_3 (13.28 mg, 0.04 mmol). After stirring at room temperature for 12 hrs (overnight), the reaction mixture was diluted with AcOEt (10 mL) and washed with water (5x5 mL), organic phase was evaporated under vacuum. Crude product was purified by PTLC (DCM:MeOH, 9:1) to give 14 mg of product (95% yield). ^1H NMR (400 MHz, DMSO-d6) δ 10.39 (s, 1H), 10.01 (s, 1H), 8.48 (d, J = 5.2 Hz, 1H), 7.90 (d, J = 13.2 Hz, 1H), 7.70 – 7.59 (m, 2H), 7.59 – 7.47 (m, 2H), 7.48 – 7.34 (m, 2H), 7.15 (t, J = 8.8 Hz, 2H), 6.43 (d, J = 5.1 Hz, 1H), 4.27 (t, J = 9.2 Hz, 1H), 3.96 (s, 3H), 3.84 (t, J = 4.2 Hz, 2H), 3.70 – 3.48 (m, 6H), 2.42 (t, J = 6.2 Hz, 2H), 1.48 (s, 4H), 1.38 (d, J = 1.6 Hz, 9H). ^{13}C NMR (151 MHz, DMSO-d6) δ 170.39 , 168.29 , 167.91 , 159.32 , 158.30 (d, J = 240.2 Hz), 153.27 (d, J = 245.6 Hz), 151.81 , 149.48 , 148.78 , 146.29 , 138.01 (d, J = 9.8 Hz), 135.66 (d, J = 12.5 Hz), 135.17 (d, J = 2.5 Hz), 123.78 , 122.44 (d, J = 7.8 Hz), 116.90 (d, J = 3.2 Hz), 114.94 , 114.57 , 108.99 (d, J = 23.0 Hz), 108.56 , 101.96 , 99.05 , 79.70 , 69.90 , 68.69 , 68.03 , 66.26 , 55.73 , 35.83 , 31.86 , 27.74 , 15.37. LC-MS (ESI); m/z: $[M+H]^+$ Calcd. for $C_{38}H_{42}F_2N_3O_9$, 722.2889 . Found 722.3494.

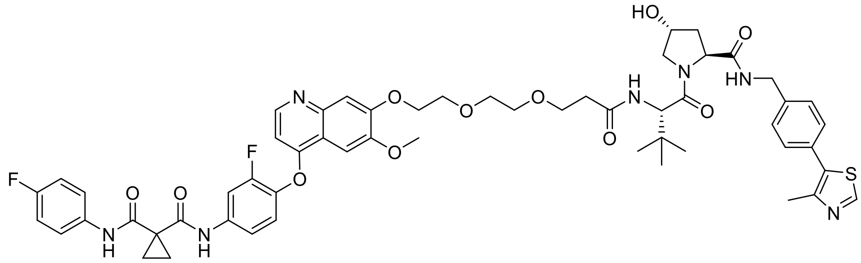

N-(3-fluoro-4-((7-(2-(2-(3-(((S)-1-((2S,4R)-4-hydroxy-2-((4-(4-methylthiazol-5-yl)benzyl) - carba-moyl)pyrrolidin-1-yl)-3,3-dimethyl-1-oxobutan-2-yl)amino)-3-oxopropoxy)ethoxy) - ethoxy)-6-methoxyquinolin-4-yl)oxy)phenyl)-N-(4-fluorophenyl)cyclopropane-1,1-dicarbo - xamide (PROTAC SJF-0693). A solution of tert-butyl ester **(14)** (13.5 mg, 0.02 mmol) in a mixture of TFA (0.7 ml, 9.42 mmol) and Dichloromethane (2 ml) was stirred for 1 h. Then the solvent was removed under vacuum and crude product was dried under high vacuum for 2 h. Crude

product was used in the next step without any further purification (12.4 mg, quantitative yield). LC-MS (ESI); m/z: $[M+H]^+$ Calcd. for $C_{34}H_{34}F_2N_3O_9$, 666.2263. Found 666.2394.

To a solution of 3-[2-[2-[[4-[2-fluoro-4-[[1-[(4-fluorophenyl)carbamoyl]cyclopropane - carbonyl]amino]phenoxy]-6-methoxy-7-quinolyl]oxy]ethoxy]ethoxy]propanoic acid from above (12.45 mg, 0.02 mmol) and (2S,4R)-1-[(2S)-2-amino-3,3-dimethyl-butanoyl]-4-hydroxy-N- [[4-(4-methylthiazol-5-yl)phenyl]methyl]pyrrolidine-2-carboxamide;hydrochloride **(12)**[3] (10.48 mg, 0.02 mmol) in N,N-Dimethylformamide (2 ml) was added N,N-Diisopropylethylamine (0.25 ml, 1.41 mmol) and O-(7-Azabenzotriazol-1-yl)-N,N,N',N'-tetramethyluronium hexafluorophosphate (10.67 mg, 0.03 mmol) at room temperature. The reaction mixture was stirred for 12 h (overnight) at the same temperature. TLC (DCM:MeOH:NH_4OH, 90:9:1) shows no starting materials. Reaction mixture was diluted with ACOEt (10 mL), washed with water (4x10 mL), dried (Na_2SO_4) and evaporated under vacuum. Crude product was purified by PTLC (DCM:MeOH:NH_4OH, 90:9:1), to give 16.5 mg of product (81 % yield). ^1H NMR (500 MHz, DMSO-d6) δ 10.39 (s, 1H), 10.01 (s, 1H), 8.97 (s, 1H), 8.61 (t, J = 5.9 Hz, 1H), 8.47 (d, J = 5.2 Hz, 1H), 8.03 – 7.84 (m, 2H), 7.64 (dd, J = 8.6, 5.2 Hz, 2H), 7.55 – 7.48 (m, 2H), 7.47 – 7.30 (m, 6H), 7.15 (t, J = 8.8 Hz, 2H), 6.42 (d, J = 5.2 Hz, 1H), 5.15 (d, J = 3.2 Hz, 1H), 4.56 (d, J = 9.4 Hz, 1H), 4.49 – 4.39 (m, 2H), 4.38 – 4.33 (m, 1H), 4.31 – 4.17 (m, 3H), 3.95 (s, 3H), 3.84 (t, J = 4.1 Hz, 2H), 3.73 – 3.48 (m, 8H), 2.60 – 2.52 (m, 1H), 2.43 (s, 3H), 2.38 (dt, J = 14.3, 6.0 Hz, 1H), 2.10 – 2.00 (m, 1H), 1.95 – 1.87 (m, 1H), 1.61 – 1.40 (m, 4H), 0.93 (s, 9H). ^{13}C NMR (151 MHz, DMSO-d6) δ 171.99 , 170.02 , 169.57 , 168.32 , 167.94 , 159.37 , 158.32 (d, J = 240.2 Hz), 153.29 (d, J = 245.2 Hz), 151.85 , 151.46 , 149.51 , 148.81 , 147.73 , 146.28 , 139.51 , 138.04 (d, J = 9.9 Hz), 135.67 (d, J = 12.4 Hz), 135.21 (d, J = 2.6 Hz), 131.19 , 129.65 , 128.65 , 127.45 , 123.84 , 122.48 (d, J = 7.9 Hz), 116.95 , 115.14 , 114.99 , 109.01 (d, J = 22.8 Hz), 108.57 , 102.00 , 99.07 , 69.93 , 69.54 , 68.92 , 68.73 , 68.08 , 67.00 , 58.76 , 56.35 , 55.77 , 41.69 , 37.97 , 35.70 , 35.40 , 31.93 , 26.34 , 15.96 , 15.37. LC-MS (ESI); m/z $[M+H]^+$: Calcd. for $C_{56}H_{62}F_2N_7O_{11}S$, 1078.4196. Found 1078.4576.

Scheme 6.- Synthesis of PROTAC **SJF-6696**

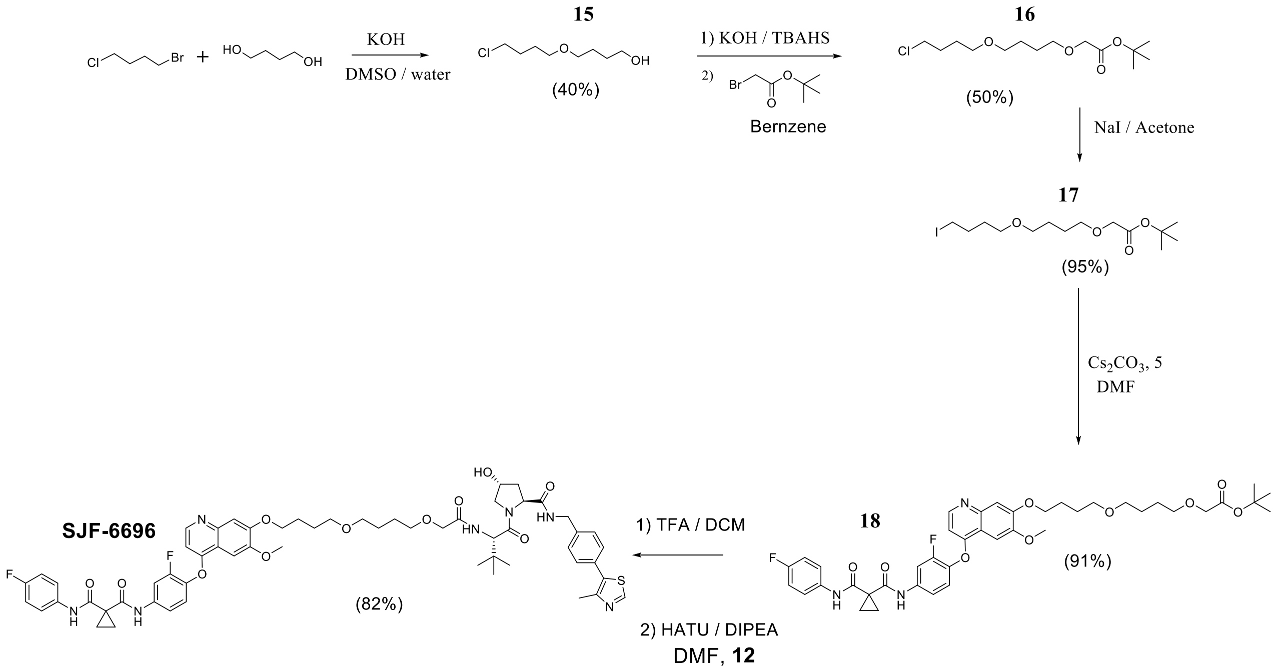

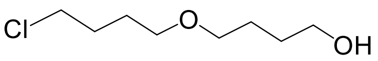

4-(4-Chlorobutoxy)butan-1-ol (15). A solution of butane-1,4-diol (23.14 ml, 260.35 mmol) and KOH (2.43 g, 43.39 mmol) in a mixture of DMSO (135 ml) and distilled water (15 mL) was stirred for 15 min at rt. Then the mixture was cooled down to 0 °C and 1-bromo-4-chloro-butane (5 ml, 43.39 mmol) was added dropwise over 1 h. The resulting solution was stirred 1 h at 0 °C and then 12 h (overnight) at rt. After dilution with a mixture of ether/hexane (1:1, 250 ml) and water (250 mL), the organic phase was separated. Water layer was extracted with a mixture ether/hexane (2x150 mL). Organic extracts were combined and washed with water (4x150 mL), dried over Na_2SO4, and concentrated under vacuum. Crude product was subjected to flash column chromatography (SiO_2-120 g, gradient of Hexane 100% to Hexane :AcOEt, 1:1 in 20 minutes) to give 3.18 g (40% yield) of product as an oil. ^{1}H NMR (400 MHz, Chloroform-d) δ 3.64 (t, J = 5.7 Hz, 2H), 3.57 (t, J = 6.5 Hz, 2H), 3.51 – 3.43 (m, 4H), 2.15 (s, 1H), 1.91 – 1.81 (m, 2H), 1.79 – 1.61 (m, 6H). ^{13}C NMR (151 MHz, Chloroform-d) δ 71.09, 70.24, 62.92, 45.06, 30.46, 29.60, 27.13, 27.02. LC-MS (ESI); m/z: $[M+H]^+$ Calcd. for $C_8H_{18}ClO_2$, 181.0995. Found 181.0990.

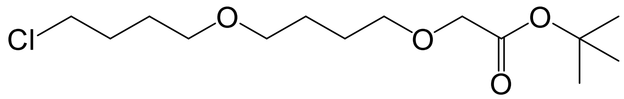

tert-Butyl 2-(4-(4-chlorobutoxy)butoxy)acetate (16). To a solution of 4-(4-chlorobutoxy)butan-1-ol **(15)** (557 mg, 3.08 mmol) and tert-butyl 2-bromoacetate (0.91 ml, 6.17 mmol) in benzene (12 mL) was added aqueous 50% NaOH (2.47 ml, 30.83 mmol) and TBAHS (1046.76 mg, 3.08 mmol). The reaction mixture was stirred vigorously at room temperature for 4 h. Then the reaction was diluted with EtOAc (50 mL) and water (50 mL), organic layer was separated, washed with water (2x50 mL), dried (Na_2SO_4) and evaporated under vacuum. Crude product was purified by flash column chromatography (SiO_2-80 g, gradient; Hex 100% to Hexane:AcOEt, 80:20 in 40 minutes) to give 543 mg of product (60 % yield) as an oil. ^{1}H NMR (400 MHz, Chloroform-d) δ 3.94 (s, 2H), 3.55 (dt, J = 13.2, 6.4 Hz, 4H), 3.43 (t, J = 6.2 Hz, 4H), 1.92 – 1.78 (m, 2H), 1.78 – 1.60 (m, 6H), 1.48 (s, 9H). ^{13}C NMR (151 MHz, Chloroform-d) δ 169.95, 81.61, 71.58, 70.71, 69.99, 68.90, 45.17, 29.71, 28.27, 27.25, 26.57, 26.46. LC-MS (ESI): m/z;$[M+Na]^+$ Calcd. for $C_{14}H_{27}ClO_4Na$:317.1495, Found: 317.1446.

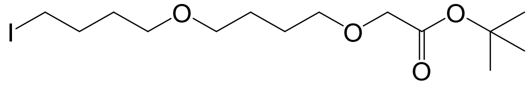

tert-Butyl 2-(4-(4-iodobutoxy)butoxy)acetate (17). To a solution of tert-butyl 2-[4-(4-chlorobutoxy)butoxy]acetate **(16)** (97 mg, 0.33 mmol) in Acetone (15 ml) was added NaI (493.18 mg, 3.29 mmol). The reaction mixture was stirred at reflux temperature for 24 h, then the solvent was removed under vacuum and crude product was dissolved in EtOAc (15 mL) and an aqueous solution of Na_2SO_3 (10%, 10 mL), organic layer was separated, washed with water (10 mL), dried (Na_2SO_4) and evaporated under vacuum. Crude product was pure by NMR (>95% purity, 121 mg, 95% yield) It was used in the next step without any further purification; ^{1}H NMR (400 MHz, Chloroform-d) δ 3.95 (s, 2H), 3.53 (t, J = 6.2 Hz, 2H), 3.42 (t, J = 6.2 Hz, 4H), 3.22 (t, J = 7.0 Hz, 2H), 1.98 – 1.84 (m, 2H), 1.74 – 1.59 (m, 6H), 1.48 (s, 9H). ^{13}C NMR (151 MHz, Chloroform-d) δ 169.96, 81.62, 71.59, 70.74, 69.66, 68.92, 30.78, 30.60, 28.28, 26.57, 26.47, 7.11. LC-MS (ESI): m/z;$[M+Na]^+$ Calcd. for $C_{14}H_{27}IO_4Na$: 409.0851, Found: 409.0808.

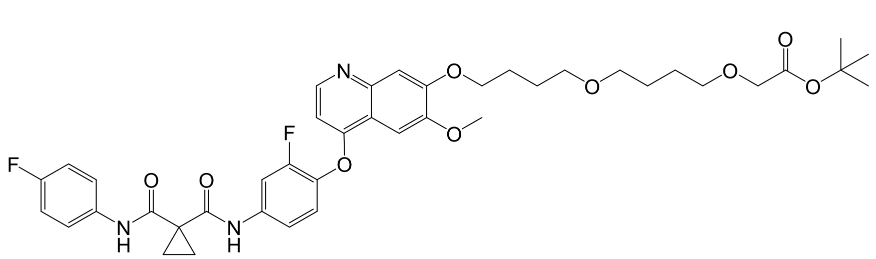

tert-Butyl 2-(4-(4-((4-(2-fluoro-4-(1-((4-fluorophenyl)carbamoyl)cyclopropane-1-carboxa-mido)-phenoxy)-6-methoxyquinolin-7-yl)oxy)butoxy)butoxy)acetate (18). To a mixture of compound **(5)**[2] and tert-butyl 2-[4-(4-iodobutoxy)butoxy]acetate **(17)** (27.51 mg, 0.07 mmol) in N,N-Dimethylformamide (2 mL) was added Cs_2CO_3 (23.2 mg, 0.07 mmol). After stirring at room

temperature for 4 hrs, the reaction mixture was diluted with AcOEt (10 mL) and washed with water (5x5 mL), organic phase was evaporated under vacuum. Crude product was purified by PTLC (DCM:MeOH:NH_4OH, 90:9:1) to give 25 mg of product (91% yield). ^1H NMR (400 MHz, DMSO-d6) δ 10.38 (s, 1H), 10.01 (s, 1H), 8.46 (d, J = 5.2 Hz, 1H), 7.90 (d, J = 13.3 Hz, 1H), 7.64 (dd, J = 8.5, 5.2 Hz, 2H), 7.57 – 7.46 (m, 2H), 7.45 – 7.36 (m, 2H), 7.15 (t, J = 8.8 Hz, 2H), 6.41 (d, J = 5.1 Hz, 1H), 4.17 (t, J = 6.3 Hz, 2H), 3.95 (s, 3H), 3.92 (s, 2H), 3.49 – 3.35 (m, 6H), 1.86 (p, J = 6.8 Hz, 2H), 1.69 (p, J = 6.5 Hz, 2H), 1.60 – 1.50 (m, 4H), 1.51 – 1.43 (m, 4H), 1.40 (s, 9H). ^{13}C NMR (151 MHz, DMSO-d6) δ 169.52 , 168.31 , 167.93 , 159.32 , 158.31 (d, J = 240.0 Hz), 153.29 (d, J = 245.1 Hz), 151.96 , 149.59 , 148.81 , 146.42 , 138.02 (d, J = 9.9 Hz), 135.70 (d, J = 12.3 Hz), 135.20 (d, J = 2.6 Hz), 123.83 , 122.47 (d, J = 7.9 Hz), 116.93 (d, J = 3.3 Hz), 115.13 , 114.98 , 114.44 , 109.00 (d, J = 22.7 Hz), 108.47 , 101.93 , 98.98 , 80.58 , 70.37 , 69.72 , 69.58 , 68.17 , 67.99 , 55.79 , 31.91 , 27.76 , 26.04 , 25.99 , 25.94 , 25.48 , 15.36. LC-MS (ESI); m/z: $[M+H]^+$ Calcd. for $C_{41}H_{48}F_2N_3O_9$.

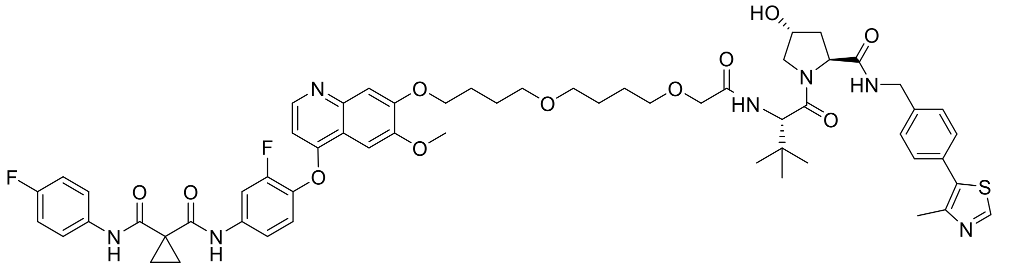

N-(3-Fluoro-4-((7-(4-(4-(2-(((S)-1-((2S,4R)-4-hydroxy-2-((4-(4-methylthiazol-5-yl)benzyl) carbamoyl)pyrrolidin-1-yl)-3,3-dimethyl-1-oxobutan-2-yl)amino)-2-oxoethoxy)butoxy)buto -xy)-6-methoxyquinolin-4-yl)oxy)phenyl)-N-(4-fluorophenyl)cyclopropane-1,1-dicarboxa- mide (PROTAC SJF-6696). A solution of tert-butyl 2-[4-[4-[[4-[2-fluoro-4-[[1-[(4-fluoro- phenyl)carbamoyl]cyclo -propanecarbonyl]amino]phenoxy]-6-methoxy-7-quinolyl]oxy]butoxy] - butoxy]acetate **(18)** (10 mg, 0.01 mmol) in a mixture of TFA (0.7 ml, 9.42 mmol) and DCM (2 ml) was stirred for 1 h. Then the solvent was removed under vacuum and crude product was dried under high vacuum for 2 h. Crude product was used in the next step without any further purification (9.2 mg, quantitative yield). LC-MS (ESI); m/z: $[M+H]^+$ Calcd. for $C_{37}H_{40}F_2N_3O_9$, 708.2732. Found 708.2838.

To a solution of 2-[4-[4-[[4-[2-fluoro-4-[[1-[(4-fluorophenyl)carbamoyl]cyclopropanecarbonyl] - amino]phenoxy]-6-methoxy-7-quinolyl]oxy]butoxy]butoxy]acetic acid from above (9.2 mg, 0.01 mmol) and compound **(12)**[3] (7.29 mg, 0.02 mmol) in N,N-Dimethylformamide (2 ml) was added N,N-Diisopropylethylamine (0.17 ml, 0.98 mmol) and O-(7-Azabenzotriazol-1-yl)-N,N,N',N'-tetramethyluronium hexafluorophosphate (7.41 mg, 0.02 mmol) at room temperature. The reaction mixture was stirred for 12 h (overnight) at the same temperature. TLC (DCM:MeOH:NH_4OH, 90:9:1) shows no starting materials. Reaction mixture was diluted with ACOEt (10 mL), washed with water (4x10 mL), dried (Na_2SO_4) and evaporated under vacuum. Crude product was purified by PTLC (DCM:MeOH:NH_4OH, 90:9:1) to give 12 mg of product (82 % yield). ^1H NMR (400 MHz, DMSO-d6) δ 10.38 (s, 1H), 10.00 (s, 1H), 8.95 (s, 1H), 8.61 (t, J = 6.0 Hz, 1H), 8.44 (d, J = 5.2 Hz, 1H), 7.88 (dd, J = 13.2, 2.1 Hz, 1H), 7.63 (dd, J = 9.1, 5.1 Hz, 2H), 7.55 – 7.45 (m, 2H), 7.44 – 7.28 (m, 6H), 7.13 (t, J = 8.9 Hz, 2H), 6.39 (d, J = 4.8 Hz, 1H), 5.15 (d, J = 3.5 Hz, 1H), 4.54 (d, J = 9.6 Hz, 1H), 4.48 – 4.30 (m, 3H), 4.23 (dd, J = 15.8, 5.5 Hz, 2H), 4.12 (t, J = 6.4 Hz,

2H), 3.92 (s, 3H), 3.90 (s, 2H), 3.68 – 3.55 (m, 2H), 3.51 – 3.34 (m, 5H), 2.41 (s, 3H), 2.12 – 2.00 (m, 1H), 1.93 – 1.76 (m, 3H), 1.72 – 1.60 (m, 2H), 1.61 – 1.52 (m, 4H), 1.50 – 1.41 (m, 4H), 0.91 (s, 9H). ^{13}C NMR (126 MHz, DMSO-d6) δ 172.20 , 169.57 , 168.92 , 168.73 , 168.35 , 159.75 , 158.72 (d, J = 240.3 Hz), 153.69 (d, J = 245.4 Hz), 152.39 , 151.85 , 150.01 , 149.20 , 148.16 , 146.79 , 139.86 , 138.43 (d, J = 10.3 Hz), 136.11 (d, J = 12.5 Hz), 135.61 (d, J = 2.6 Hz), 131.57 , 130.12 , 129.10 , 127.90 , 124.23 , 122.88 (d, J = 7.7 Hz), 117.36 , 115.47 (d, J = 22.2 Hz), 114.86 , 109.42 (d, J = 22.8 Hz), 108.86 , 102.35 , 99.40 , 70.14 , 70.01 , 69.83 , 69.33 , 68.58 , 59.20 , 57.05 , 56.21 , 56.06 , 42.12 , 38.33 , 36.30 , 32.34 , 26.59 , 26.46 , 26.42 , 26.35 , 25.87 , 16.34 , 15.78. LC-MS (ESI); m/z [M+H]$^+$: Calcd. for $C_{59}H_{68}F_2N_7O_{11}S$, 1120.4665. Found 1120.5044.

Scheme 7.- Synthesis of PROTAC **SJF-8240**

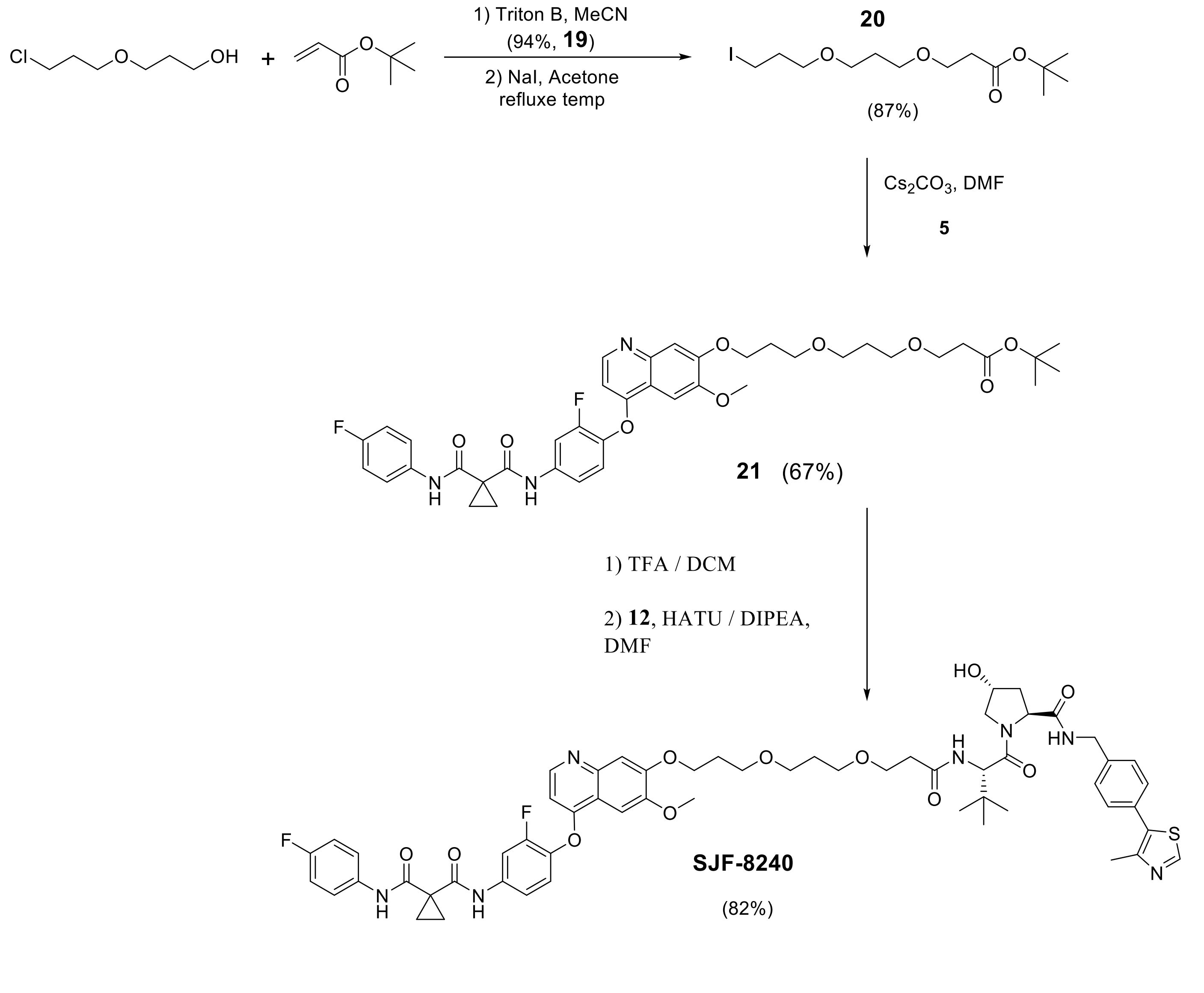

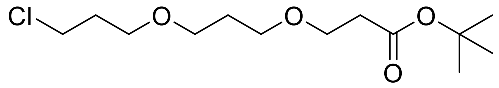

***tert*-Butyl 3-(3-(3-chloropropoxy)propoxy)propanoate (19).** 3-(3-chloropropoxy)propan-1-ol (66 mg, 0.43 mmol) in acetonitrile (3 mL) was added tert-butyl prop-2-enoate (0.31 ml, 2.16 mmol) followed by Triton B (54 mg, 0.1 mmol, 40% by weight in water). The mixture was stirred at room temperature for 72 hour. The mixture was concentrated under vacuum and crude product was purified by column chromatography (SiO_2, gradient Hex:EtOAc, 95:5 to 9:1) to give 115 mg of product (**19**) as an oil (94% yield). ^{1}H NMR (500 MHz, Chloroform-d) δ 3.70 – 3.59 (m, 4H), 3.59 – 3.42 (m, 6H), 2.47 (t, J = 6.5 Hz, 2H), 2.04 – 1.96 (m, 2H), 1.82 (p, J = 6.3 Hz, 2H), 1.45 (s, 9H). ^{13}C NMR (151 MHz, Chloroform-d) δ 171.13, 80.63, 68.02, 67.97, 67.27, 66.64, 42.17, 36.50, 32.88, 30.09, 28.25. LC-MS (ESI); m/z [M+Na]$^+$: Calcd. for $C_{13}H_{25}ClO_4Na$, 303.1339. Found 303.1381 for ^{35}(Cl), and m/z [M+2+Na]$^+$ 305.1385, for ^{37}(Cl).

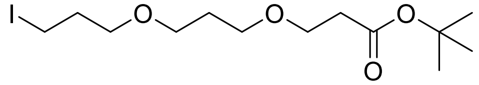

***tert*-Butyl 3-(3-(3-iodopropoxy)propoxy)propanoate (20)**. To a solution of tert-butyl 3-[3-(3-chloropropoxy)propoxy]propanoate **(19)** (161 mg, 0.57 mmol) in Acetone (5 ml) was added NaI (429 mg, 2.87 mmol). The reaction mixture was stirred at reflux temperature for 24 h, then the solvent was removed under vacuum and crude product was dissolved in EtOAc (15 mL), washed with water (10 mL), and with an aqueous solution of Na_2SO_3 (10%, 10 mL). Organic layer was separated, washed with water (10 mL), dried (Na_2SO_4) and evaporated under vacuum. Crude product was pure by NMR (>98% purity, 186 mg, 87% yield), product (**20**) was used in the next step without any further purification. ^{1}H NMR (400 MHz, Chloroform-d) δ 3.66 (t, J = 6.5 Hz, 2H), 3.57 – 3.40 (m, 6H), 3.27 (t, J = 6.8 Hz, 2H), 2.48 (t, J = 6.5 Hz, 2H), 2.08 – 1.99 (m, 2H), 1.82 (p, J = 6.4 Hz, 2H), 1.45 (s, 9H). ^{13}C NMR (151 MHz, Chloroform-d) δ 171.13, 80.64, 70.18, 68.01, 67.98, 66.65, 36.50, 33.57, 30.10, 28.26, 3.72. LC-MS (ESI): m/z [M+Na]$^+$ Calcd. for $C_{13}H_{25}IO_4Na$: 395.0695, Found: 395.0719.

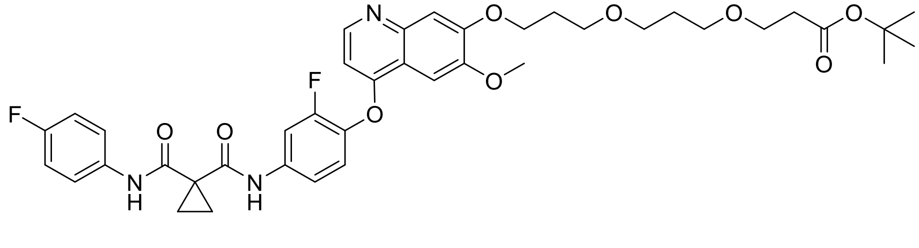

tert-Butyl 3-(3-(3-((4-(2-fluoro-4-(1-((4-fluorophenyl)carbamoyl)cyclopropane-1-carboxamido)-phenoxy)-6-methoxyquinolin-7-yl)oxy)propoxy)propoxy)propanoate (**21**). To a mixture of N1'-[3-fluoro-4-[(7-hydroxy-6-methoxy-4-quinolyl)oxy]phenyl]-N1-(4-fluoro-

phenyl)cyclopropane -1,1-dicarboxamide (**5**)[2] (15 mg, 0.03 mmol) and tert-butyl 3-[3-(3-iodopropoxy)propoxy]propanoate (**20**) (16.57 mg, 0.04 mmol) in N,N-Dimethylformamide (1 mL) was added Cs_2CO_3 (29.01 mg, 0.09 mmol). After stirring at room temperature for 12 hrs (overnight), the reaction mixture was diluted with AcOEt (20 mL) and washed with water (5x10 mL), organic phase was evaporated under vacuum. Crude product was purified by PTLC (DCM:MeOH:NH_4OH, 92:7:1) to give 15 mg of product (**21**) (67% yield). ^1H NMR (400 MHz, DMSO-d6) δ 10.39 (s, 1H), 10.01 (s, 1H), 8.46 (d, J = 5.2 Hz, 1H), 7.90 (d, J = 13.2 Hz, 1H), 7.71 – 7.58 (m, 2H), 7.51 (d, J = 7.4 Hz, 2H), 7.46 – 7.35 (m, 2H), 7.15 (t, J = 8.9 Hz, 2H), 6.41 (d, J = 5.1 Hz, 1H), 4.21 (t, J = 6.2 Hz, 2H), 3.95 (s, 3H), 3.60 – 3.37 (m, 8H), 2.37 (d, J = 12.2 Hz, 2H), 2.04 (p, J = 6.4 Hz, 2H), 1.71 (p, J = 6.4 Hz, 2H), 1.47 (s, 4H), 1.37 (s, 9H). ^{13}C NMR (151 MHz, dmso) δ 170.45, 168.27, 167.87, 159.29, 158.28 (d, J = 240.2 Hz), 153.26 (d, J = 245.1 Hz), 151.89, 149.56, 148.82, 146.37, 138.02 (d, J = 9.9 Hz), 135.66 (d, J = 12.4 Hz), 135.20 (d, J = 2.7 Hz), 123.82, 122.43 (d, J = 7.9 Hz), 116.90, 115.04 (d, J = 22.2 Hz), 114.47, 108.96 (d, J = 23.1 Hz), 108.50, 101.95, 99.01, 79.64, 67.07, 66.55, 65.92, 65.45, 55.79, 35.87, 31.93, 29.53, 28.90, 27.76, 27.73, 15.31. LC-MS (ESI): m/z $[M+H]^+$ Calcd. for $C_{40}H_{46}F_2N_3O_9$, 750.3202 . Found 750.3509.

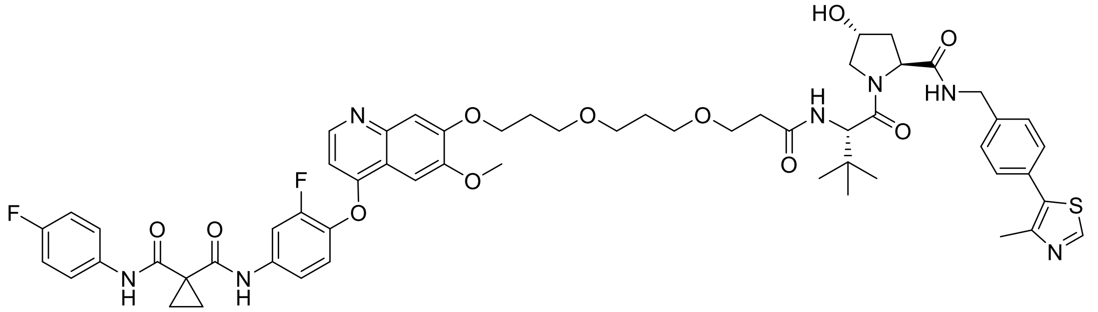

N-(3-Fluoro-4-((7-(3-(3-(3-(((S)-1-((2S,4R)-4-hydroxy-2-((4-(4-methylthiazol-5-yl)benzyl)carbamoyl)-pyrrolidin-1-yl)-3,3-dimethyl-1-oxobutan-2-yl)amino)-3-oxopropoxy)propoxy)propoxy)-6-methoxyquinolin-4-yl)oxy)phenyl)-N-(4-fluorophenyl)cyclopropane-1,1-dicarboxamide (PROTAC SJF-8240). A solution of 3-[3-[3-[[4-[2-fluoro-4-[[1-[(4-fluorophenyl)carbamoyl] cyclopropanecarbonyl]amino]phenoxy]-6-methoxy-7-quinolyl]oxy]propoxy]propoxy]propanoic acid (**21**) (15 mg, 0.02 mmol) in a mixture of TFA (1 ml, 13.46 mmol) and Dichloromethane (3 ml) was stirred for 2 h. Then the solvent was removed under vacuum and crude product was dried under high vacuum for 2 h. Crude product was used in the next step without any further purification (13.8 mg, quantitative yield). LC-MS (ESI): m/z [M+H]+ Calcd. for $C_{36}H_{38}F_2N_3O_9$, 694.2576. Found 694.2324.

To a solution of crude product from above (13.8 mg, 0.02 mmol) and (2S,4R)-1-[(2S)-2-amino -3,3-dimethyl-butanoyl]-4-hydroxy-N-[[4-(4-methylthiazol-5-yl)phenyl]methyl]-pyrrolidine-2-carboxamide;hydrochloride **(12)**[3] (11.15 mg, 0.02 mmol) in N,N-Dimethylformamide (2 ml) was added DIPEA (0.17 ml, 0.99 mmol) and HATU (11.35 mg, 0.03 mmol) at room temperature. The reaction mixture was stirred for 12 h (overnight) at the same temperature. Reaction mixture was diluted with ACOEt (20 mL), washed with water (4x15 mL), dried (Na_2SO_4) and evaporated under vacuum. Crude product was purified by PTLC (DCM:MeOH:NH_4OH, 90:9:1), to give 18 mg of product (82 % yield). ^1H NMR (500 MHz, DMSO-d6) δ 10.38 (s, 1H), 10.00 (s, 1H), 8.97 (s, 1H), 8.56 (t, J = 6.1 Hz, 1H), 8.46 (d, J = 5.2 Hz, 1H), 7.96 – 7.85 (m, 2H), 7.69 – 7.59 (m, 2H), 7.51

(d, J = 8.8 Hz, 2H), 7.45 – 7.33 (m, 5H), 7.15 (t, J = 8.9 Hz, 2H), 6.41 (d, J = 5.1 Hz, 1H), 5.12 (d, J = 3.3 Hz, 1H), 4.55 (d, J = 9.4 Hz, 1H), 4.43 (ddd, J = 10.9, 6.7, 3.3 Hz, 2H), 4.27 – 4.16 (m, 3H), 3.94 (s, 3H), 3.76 – 3.33 (m, 10H), 2.58 – 2.51 (m, 1H), 2.43 (s, 3H), 2.35 – 2.25 (m, 1H), 2.03 (p, J = 5.7 Hz, 3H), 1.95 – 1.83 (m, 1H), 1.72 (p, J = 6.4 Hz, 2H), 1.48 (d, J = 3.9 Hz, 4H), 0.92 (s, 9H). ^{13}C NMR (126 MHz, dmso) δ 171.89, 169.97, 169.51, 168.26, 167.88, 159.31, 158.27 (d, J = 240.1 Hz), 153.23 (d, J = 245.1 Hz), 151.90, 151.39, 149.56, 148.75, 147.69, 146.29, 139.47, 137.97 (d, J = 9.8 Hz), 135.65 (d, J = 12.4 Hz), 135.16 (d, J = 2.5 Hz), 131.13, 129.61, 128.61, 127.40, 123.77, 122.43 (d, J = 7.9 Hz), 116.90, 115.00 (d, J = 22.2 Hz), 114.47, 108.96 (d, J = 23.0 Hz), 108.45, 101.94, 99.03, 68.85, 67.16, 67.09, 66.62, 66.54, 65.47, 58.69, 56.35, 56.24, 55.77, 41.64, 37.92, 35.69, 35.36, 31.87, 29.60, 28.89, 26.28, 15.91, 15.31. LC-MS (ESI): m/z $[M+H]^+$ Calcd. for $C_{58}H_{66}F_2N_7O_{11}S$, 1106.4509. Found 1106.4510.

Scheme 8.- Synthesis of PROTAC **BES-4612**

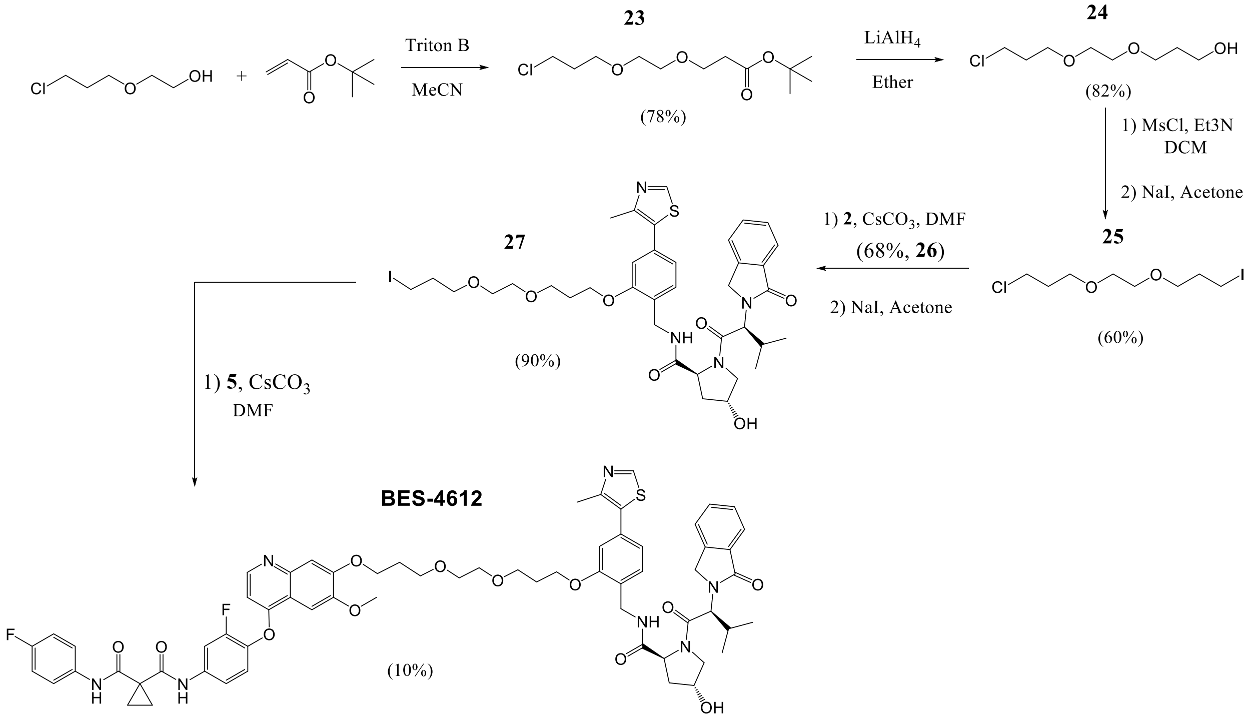

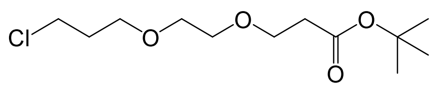

tert-Butyl 3-(2-(3-chloropropoxy)ethoxy)propanoate (23). To a solution of 2-(3-chloropropoxy) -ethan-1-ol (1.48 g, 10.68 mmol) in acetonitrile (20 mL) was added tert-butyl prop-2-enoate (7.75 ml, 128.17 mmol) followed by Triton B (447 mg, 1.06 mmol, in 40% by weight in water). The mixture was stirred at room temperature for 12 hours (overnight). The mixture was concentrated in vacuum and crude product was purified by flash chromatography (SiO_2-80g, gradient Hex:AcOEt, 98:2 to 8:2) to give 2.21 g of product as an oil (77% yield). ^1H NMR (500 MHz, Chloroform-d) δ 3.71 (t, J = 6.6 Hz, 2H), 3.63 (t, J = 6.4 Hz, 2H), 3.62 – 3.54 (m, 8H), 2.50 (t, J = 6.6 Hz, 2H), 2.02 (p, J = 6.2 Hz, 2H), 1.44 (s, 9H). ^{13}C NMR (126 MHz, cdcl3) δ 171.02, 80.66, 77.36, 70.46, 67.79, 67.08, 42.09, 36.44, 32.86, 28.25. LC-MS (ESI); m/z: $[M+Na]^+$ Calcd. for $C_{12}H_{23}ClO_4Na$, 289.1182. Found 289.1364.

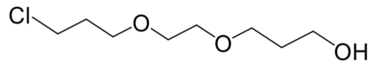

3-(2-(3-Chloropropoxy)ethoxy)propan-1-ol (24). To a solution of the ester **(23)** (1.21 g, 4.54 mmol) in diethyl ether (30 ml) was added 1M $LiAlH_4$ (6.8 ml) at 0 °C., the reaction mixture was stirred at the same temperature for 30 min., then ice-bath was removed and stirred for 30 min at room temperature. Then $Na_2SO_4 \cdot 10H_2O$ was added and the mixture was stirred for an additional 30 min. Reaction mixture was filtered under vacuum over a celite pad, and filtrate was evaporated at 50 °C (oil bath). Crude product was purified by flash chromatography (SiO_2-40g, gradient Hex:EtOAc, 2% to 1:1 in 20 min) to give 0.732 g of product as an oil (82% yield). ^1H NMR (400 MHz, Chloroform-d) δ 3.77 (t, J = 5.5 Hz, 2H), 3.72 – 3.50 (m, 10H), 2.02 (p, J = 6.2 Hz, 2H), 1.83 (p, J = 5.7 Hz, 2H). ^{13}C NMR (151 MHz, cdcl3) δ 70.56, 70.29, 70.23, 67.67, 61.96, 42.02, 32.69, 31.94. LC-MS m/z: $[M+H]^+$ Calcd. for $C_8H_{18}ClO_3$, 197.0944. Found 197.1085.

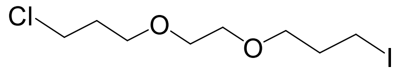

1-Chloro-3-(2-(3-iodopropoxy)ethoxy)propane (25). To a solution of 3-(2-(3-chloro-propoxy)ethoxy)propan-1-ol **(24)** (200 mg, 1.02 mmol) in Dichloromethane (5 ml) was added TEA (0.42 ml, 3.05 mmol), then reaction mixture was cooled to 0 °C (water ice/acetone bath) and mesyl chloride (0.09 ml, 1.22 mmol) was added dropwise. The reaction mixture was stirred for 1 h at the same temperature. By TLC no starting material (Hex:AcOEt, 3:7). Reaction mixture was poured into an aqueous solution of $NaHCO_3$ (20 mL) and product extracted with DCM (20mL, 2x), the organic extracts were combined, dried (Na_2SO_4), and evaporated under vacuum. The crude product (mesylate) was used in the next step without any further purification (>95% pure by NMR)): ^1H NMR (500 MHz, Chloroform-d) δ 4.35 (t, J = 6.2 Hz, 2H), 3.64 (t, J = 6.4 Hz, 2H), 3.62 – 3.53 (m, 8H), 3.01 (s, 3H), 2.02 (pd, J = 6.1, 3.8 Hz, 4H).

Crude mixture from previous step was diluted in acetonitrile (5 ml) and NaI (228.65 mg, 1.53

mmol) was added, the reaction mixture was stirred at 70 °C for 3 h. By TLC (Hex:AcOEt, 7:3) and NMR small amount of masylate, the reaction was poured into an aqueous solution of $Na_2S_2O_3$ (10%, 20 mL) and product was extracted with DCM (2x20 mL). Organic extracts were combined, dried (NA_2SO_4) and evaporated under vacuum. Crude product was purified by flash chromatography (SiO_2-25g, grad. Hex:AcOEt, 2 to 20% in 15 min), to give 187 mg of product as an oil (60% yield), small amount of bis-iodo compound by LC-MS (~10%). ^{1}H NMR (400 MHz, Chloroform-d) δ 3.65 (t, J = 6.4 Hz, 2H), 3.63 – 3.56 (m, 6H), 3.53 (t, J = 5.9 Hz, 2H), 3.28 (t, J = 6.7 Hz, 2H), 2.11 – 1.98 (m, 4H).^{13}C NMR (151 MHz, cdcl3) δ 70.67, 70.46, 70.44, 67.76, 42.12, 33.47, 32.81, 3.63. LC-MS (ESI); m/z: $[M+H]^+$ Calcd. for $C_8H_{17}ClIO_2$, 306.9961. Found 307.1626.

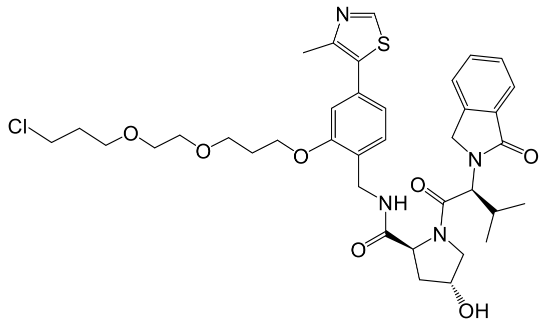

(2S,4R)-N-(2-(3-(2-(3-chloropropoxy)ethoxy)propoxy)-4-(4-methylthiazol-5-yl)benzyl)-4-hydroxy-1-((S)-3-methyl-2-(1-oxoisoindolin-2-yl)butanoyl)pyrrolidine-2-carboxamide (26). To a mixture of (2S,4R)-4-hydroxy-N-[[2-hydroxy-4-(4-methylthiazol-5-yl)phenyl]methyl]-1-[(2S)-3-methyl-2- (1-oxoisoindolin-2-yl)butanoyl]pyrrolidine-2-carboxamide **(2)**[1] (75 mg, 0.13 mmol) and 1-chloro-3-(2-(3-iodopropoxy)-ethoxy)propane **(25)** (54.48 mg, 0.18 mmol) in DMF (1 mL) was added Cs_2CO_3 (89.08 mg, 0.27 mmol). After stirring at room temperature for 2 hrs, the reaction mixture was diluted with AcOEt (10 mL) and washed with water (5x10 mL), organic phase was dried (Na_2SO_4), and evaporated under vacuum. Crude product was purified by PTLC (DCM:MEOH:NH_4OH, 90:9:1) to give 68 mg of product (68% yield): ^{1}H NMR (400 MHz, Chloroform-d) δ 8.67 (s, 1H), 7.73 (d, J = 7.7 Hz, 1H), 7.49 (td, J = 7.4, 1.2 Hz, 1H), 7.39 (dt, J = 7.5, 3.6 Hz, 2H), 7.33 – 7.23 (m, 2H), 6.95 (dd, J = 7.6, 1.6 Hz, 1H), 6.89 (s, 1H), 4.84 – 4.66 (m, 2H), 4.62 (t, J = 7.8 Hz, 1H), 4.59 – 4.28 (m, 6H), 4.12 (t, J = 6.1 Hz, 2H), 3.81 – 3.63 (m, 4H), 3.63 – 3.49 (m, 6H), 3.41 (bs, 1H), 2.52 (s, 3H), 2.50 – 2.29 (m, 2H), 2.15 (p, J = 6.1 Hz, 2H), 2.02 – 1.89 (m, 2H), 0.91 (d, J = 6.5 Hz, 3H), 0.86 (d, J = 6.6 Hz, 3H). ^{13}C NMR (101 MHz, cdcl3) δ 170.66, 170.37, 169.59, 156.87, 150.41, 148.59, 142.17, 132.36, 131.92, 131.85, 131.65, 129.39, 128.08, 126.42, 123.88, 122.94, 121.67, 112.19, 70.43, 70.40, 70.02, 67.93, 67.73, 65.26, 58.74, 58.67, 56.10, 47.57, 42.01, 38.99, 36.04, 32.71, 29.69, 29.00, 19.15, 16.24. LC-MS (ESI); m/z: $[M+H]^+$ Calcd. for $C_{37}H_{48}ClN_4O_7S$, 727.2932. Found 727.4574.

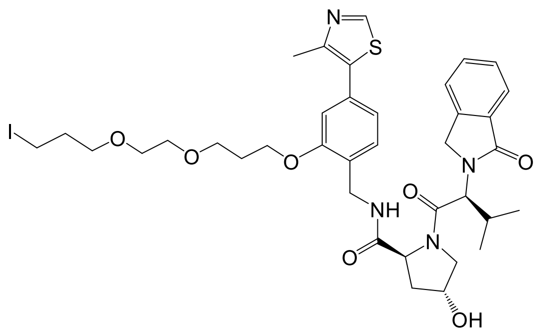

(2S,4R)-4-Hydroxy-N-(2-(3-(2-(3-iodopropoxy)ethoxy)propoxy)-4-(4-methylthiazol-5-yl)-benzyl)-1-((S)-3-methyl-2-(1-oxoisoindolin-2-yl)butanoyl)pyrrolidine-2-carboxamide (27). To a solution of (2S,4R)-N-[[2-[3-[2-(3-chloropropoxy)ethoxy]propoxy]-4-(4-methylthiazol-5-yl)phenyl]methyl]- -4-hydroxy-1-[(2S)-3-methyl-2-(1-oxoisoindolin-2-yl)butanoyl]pyrrolidine-2-carboxamide **(26)** (69 mg, 0.09 mmol) in Acetone (10 ml) was added NaI (71.1 mg, 0.47 mmol). The reaction mixture was stirred at reflux temperature for 24 h, then the solvent was removed under vacuum and crude product was dissolved in EtOAc (15 mL) and an aqueous solution of Na_2SO_3 (10%, 10 mL), organic layer was separated, washed with water (10 mL), dried (Na_2SO_4) and evaporated under vacuum. Crude product was pure by NMR (>95% purity, 70 mg, 90% yield), it was used in the next step without any further purification; ^{1}H NMR (500 MHz, Chloroform-d) δ 8.67 (s, 1H), 7.75 (dt, J = 7.7, 4.4 Hz, 1H), 7.54 – 7.47 (m, 1H), 7.44 – 7.37 (m, 2H), 7.29 (d, J = 7.7 Hz, 2H), 6.96 (d, J = 7.7 Hz, 1H), 6.90 (s, 1H), 4.80 – 4.69 (m, 2H), 4.64 (t, J = 7.8 Hz, 1H), 4.56 – 4.34 (m, 5H), 4.13 (t, J = 5.7 Hz, 2H), 3.74 – 3.62 (m, 3H), 3.64 – 3.54 (m, 4H), 3.50 (t, J = 5.9 Hz, 2H), 3.22 (t, J = 6.7 Hz, 2H), 2.52 (s, 3H), 2.52 – 2.44 (m, 1H), 2.40 (dtd, J = 13.2, 6.7, 3.4 Hz, 1H), 2.22 – 2.10 (m, 2H), 2.09 – 1.96 (m, 3H), 0.90 (d, J = 6.5 Hz, 3H), 0.87 (d, J = 6.6 Hz, 3H).^{13}C NMR (126 MHz, cdcl3) δ 170.45, 170.27, 169.49, 156.74, 150.26, 148.46, 142.05, 132.23, 131.78, 131.73, 131.54, 129.27, 127.96, 126.26, 123.77, 122.82, 121.53, 112.06, 70.51, 70.28, 69.92, 67.80, 65.12, 58.61, 58.44, 55.93, 47.43, 41.87, 38.88, 35.77, 33.23, 29.56, 28.78, 19.03, 16.13, 3.37. LC-MS (ESI): m/z;$[M+H]^+$ Calcd. for $C_{37}H_{48}IN_4O_7S$: 819.2288, Found: 819.2384.

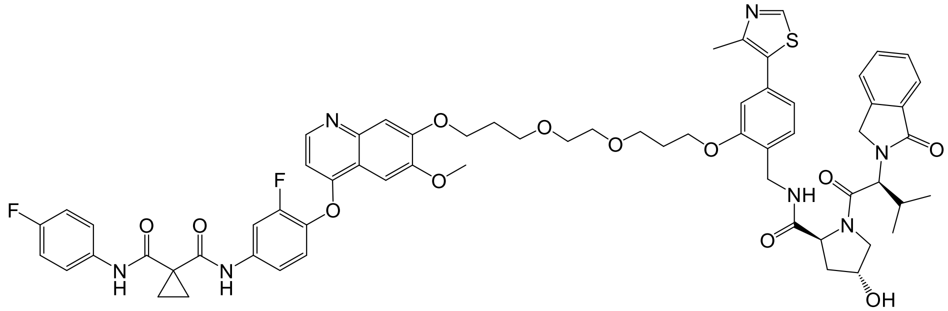

N-(3-fluoro-4-((7-(3-(2-(3-(2-(((2S,4R)-4-hydroxy-1-((S)-3-methyl-2-(1-oxoisoindolin-2-yl) -butanoyl)pyrrolidine-2-carboxamido)methyl)-5-(4-methylthiazol-5-yl)phenoxy)propoxy) -ethoxy)propoxy)-6-methoxyquinolin-4-yl)oxy)phenyl)-N-(4-fluorophenyl)cyclopropane-1,1-dicarboxamide (PROTAC BES-4612). To a mixture of (2S,4R)-4-hydroxy-N-[[2-[3-[2-(3-iodopropoxy)ethoxy]propoxy]-4-(4-methylthiazol-5-yl)phenyl]methyl]-1-[(2S)-3-methyl-2-(1-

oxoisoindolin-2-yl)butanoyl]pyrrolidine-2-carboxamide **(27)** (15.8 mg, 0.02 mmol) and N1'-[3-fluoro-4-[(7-hydroxy-6-methoxy-4-quinolyl)oxy]phenyl]-N1-(4-fluorophenyl)cyclopropane-1,1-dicarboxamide **(5)**[2] (9.75 mg, 0.02 mmol) in DMF (1 mL) was added Cs_2CO_3 (12.57 mg, 0.04 mmol). After stirring at room temperature for 12 hrs (overnight), the reaction mixture was diluted with AcOEt (10 mL) and washed with brine (5x10 mL), organic phase was dried (Na_2SO_4, and evaporated under vacuum. Crude product was purified by PTLC (DCM:MEOH:NH_4OH, 90:9:1) to give 2.5 mg of product (10% yield). ^{1}H NMR (600 MHz, DMSO-d6) δ 10.39 (s, 1H), 10.01 (s, 1H), 8.96 (s, 1H), 8.45 (d, J = 4.8 Hz, 1H), 8.38 (t, J = 5.4 Hz, 1H), 7.90 (d, J = 13.0 Hz, 1H), 7.70 (d, J = 7.5 Hz, 1H), 7.68 – 7.28 (m, 10H), 7.15 (t, J = 8.5 Hz, 2H), 7.04 – 6.92 (m, 2H), 6.40 (d, J = 5.0 Hz, 1H), 5.09 (d, J = 3.0 Hz, 1H), 4.70 (d, J = 10.8 Hz, 1H), 4.54 (d, J = 18.1 Hz, 1H), 4.49 – 4.37 (m, 2H), 4.35 – 4.26 (m, 2H), 4.23 (dd, J = 16.3, 6.0 Hz, 1H), 4.17 (t, J = 6.1 Hz, 2H), 4.09 (t, J = 5.7 Hz, 2H), 3.93 (s, 2H), 3.81 – 3.65 (m, 2H), 3.64 – 3.46 (m, 8H), 2.45 (s, 3H), 2.35 – 2.28 (m, 1H), 2.07 – 1.86 (m, 6H), 1.47 (d, J = 5.6 Hz, 4H), 0.95 (d, J = 6.2 Hz, 3H), 0.72 (d, J = 6.3 Hz, 3H). ^{13}C NMR (151 MHz, dmso) δ 171.92, 168.70, 168.50, 168.32, 167.88, 159.73, 159.50, 157.91, 156.27, 154.48, 152.86, 152.31, 151.83, 149.96, 149.20, 148.29, 146.72, 142.61, 138.46, 138.40, 136.11, 136.02, 135.62, 135.60, 131.99, 131.78, 131.68, 131.41, 128.32, 128.19, 127.41, 124.23, 124.02, 123.42, 122.89, 122.83, 121.24, 117.32, 115.53, 115.38, 114.88, 112.07, 109.46, 109.31, 108.86, 102.35, 99.43, 70.02, 70.00, 69.03, 67.32, 67.29, 65.82, 65.18, 59.10, 58.19, 56.20, 55.84, 47.22, 38.52, 37.47, 32.34, 29.51, 29.33, 28.80, 19.28, 19.03, 16.41, 15.74. LC-MS (ESI); m/z: $[M+H]^+$ Calcd. for $C_{64}H_{68}F_2N_7O_{12}S$, 1196.4614. Found 1196.4210.

Supplementary References:

1. Qian, Y. *et al*. Preparation of bifunctional PROTAC compounds and methods for the enhanced degradation of targeted bromodomain-containing proteins. WO 2017030814, (2017).

2. Bannen, L. *et al*. Preparation of quinolines and quinazolines as inhibitors of c-Met and other tyrosine kinases and therapeutic uses against proliferative diseases. WO 2005030140, (2005).

3. Galdeano, C. *et al.* Structure-guided design and optimization of small molecules targeting the protein-protein interaction between the von Hippel-Lindau (VHL) E3 ubiquitin ligase and the hypoxia inducible factor (HIF) alpha subunit with in vitro nanomolar affinities. *J Med Chem* **57**, 8657-8663, doi:10.1021/jm5011258 (2014).
