# Supplementary material for: Differential PROTAC substrate specificity dictated by orientation of recruited E3 ligase
Source: Nat Commun. 2019 Jan 10;10:131. doi: 10.1038/s41467-018-08027-7 (PMC6328587; doi:10.1038/s41467-018-08027-7)
Supplement: Supplementary file 3 — Reporting Summary [file 41467_2018_8027_MOESM3_ESM.pdf]

nature research

Corresponding author(s): Craig M. Crews, Ph.D.

Reporting Summary

Nature Research wishes to improve the reproducibility of the work that we publish. This form provides structure for consistency and transparency in reporting. For further information on Nature Research policies, see Authors & Referees and the Editorial Policy Checklist.

Statistical parameters

When statistical analyses are reported, confirm that the following items are present in the relevant location (e.g. figure legend, table legend, main text, or Methods section).

n/a	Confirmed	
☐	☒	The exact sample size (n) for each experimental group/condition, given as a discrete number and unit of measurement
☐	☒	An indication of whether measurements were taken from distinct samples or whether the same sample was measured repeatedly
☐	☒	The statistical test(s) used AND whether they are one- or two-sided<br>*Only common tests should be described solely by name; describe more complex techniques in the Methods section.*
☒	☐	A description of all covariates tested
☒	☐	A description of any assumptions or corrections, such as tests of normality and adjustment for multiple comparisons
☐	☒	A full description of the statistics including central tendency (e.g. means) or other basic estimates (e.g. regression coefficient) AND variation (e.g. standard deviation) or associated estimates of uncertainty (e.g. confidence intervals)
☐	☒	For null hypothesis testing, the test statistic (e.g. F, t, r) with confidence intervals, effect sizes, degrees of freedom and P value noted<br>*Give P values as exact values whenever suitable.*
☒	☐	For Bayesian analysis, information on the choice of priors and Markov chain Monte Carlo settings
☒	☐	For hierarchical and complex designs, identification of the appropriate level for tests and full reporting of outcomes
☒	☐	Estimates of effect sizes (e.g. Cohen's d, Pearson's r), indicating how they were calculated
☐	☒	Clearly defined error bars<br>*State explicitly what error bars represent (e.g. SD, SE, CI)*

Our web collection on statistics for biologists may be useful.

Software and code

Policy information about availability of computer code

Data collection	Intensity of immunoreactive band signal from immunoblots was measured using Image Lab software (Bio-Rad Laboratories). AlphaLISA results were collected using Gen5 imager software (BioTek Instruments).
Data analysis	Dose-response data was fit to sigmoidal curves using PRISM (GraphPad Software). Analysis of data from surface plasmon resonance experiments was performed using the program Scrubber 2 (BioLogic Software). Molecular dynamics simulations were performed using Desmond Multisim (GPU-enabled) v3.8.5.19 (Schrodinger, Inc.).

For manuscripts utilizing custom algorithms or software that are central to the research but not yet described in published literature, software must be made available to editors/reviewers upon request. We strongly encourage code deposition in a community repository (e.g. GitHub). See the Nature Research guidelines for submitting code & software for further information.

Data

Policy information about availability of data

All manuscripts must include a data availability statement. This statement should provide the following information, where applicable:

- Accession codes, unique identifiers, or web links for publicly available datasets
- A list of figures that have associated raw data
- A description of any restrictions on data availability

The authors declare that data supporting the findings of this study are available within the paper and its supplementary information files.

Field-specific reporting

Please select the best fit for your research. If you are not sure, read the appropriate sections before making your selection.

☒ Life sciences ☐ Behavioural & social sciences ☐ Ecological, evolutionary & environmental sciences

For a reference copy of the document with all sections, see nature.com/authors/policies/ReportingSummary-flat.pdf

Life sciences study design

All studies must disclose on these points even when the disclosure is negative.

Sample size	Experimental sample size was set at three replications (or greater, as in the AlphaLISA) for the purposes of statistical analysis.
Data exclusions	No data was excluded from analysis.
Replication	All attempts at replication of experimental results were successful.
Randomization	Cultured cells were passaged evenly (random distribution) into dishes for treatment with PROTACs or other biologically-active compounds. Due to dose-response nature of the experiments performed, randomization of the samples post-treatment would make data interpretation impossible.
Blinding	Investigators were not blinded to the nature of their samples during data collection and analysis.

Reporting for specific materials, systems and methods

Materials & experimental systems

n/a	Involved in the study	
☒	☐	Unique biological materials
☐	☒	Antibodies
☐	☒	Eukaryotic cell lines
☒	☐	Palaeontology
☒	☐	Animals and other organisms
☒	☐	Human research participants

Methods

n/a	Involved in the study	
☒	☐	ChIP-seq
☒	☐	Flow cytometry
☒	☐	MRI-based neuroimaging

Antibodies

Antibodies used	The following antibodies were used in this study: anti-p38alpha (Cell Signaling Technologies cat. no. 9218, lot no. 8); anti-p38delta (Cell Signaling Technologies cat. no. 2308, lot no. 2); anti-p38beta (Cell Signaling Technologies cat. no. 2339, lot no. 3); anti-p38gamma (Cell Signaling Technologies cat. no. 2307, lot no. 2); anti-ERK2 (Cell Signaling Technologies cat. no. 9108, lot no. 6); anti-JNK2 (Cell Signaling Technologies cat. no. 9258, lot no. 11); anti-VHL (Cell Signaling Technologies cat. no. 68547, lot no. 1); anti-HA (Cell Signaling Technologies cat. no. 2367, lot no. 5); anti-JNK1 (Cell Signaling Technologies cat. no. 3708, lot no. 3); anti-FLAG sepharose (Cell Signaling Technologies cat. no. 70569, lot no. 1); anti-tubulin (Sigma-Aldrich cat. no. T9026, lot no. 047M4789V); anti-FLAG M2 (Sigma-Aldrich cat. no. F1804, lot no. SLBV9325); anti-cullin 2 (ThermoFisher cat. no. 700179, lot no. 1448733B); and anti-ERK1 (Santa Cruz Biotechnology cat. no. sc-93, lot B1512).
Validation	All the antibodies used in this study were purchased from commercial sources and had been validated by their respective manufacturer for immunoblotting and/or immunoprecipitation (the applications used in this study) of the human isoform of

their cognate antigens.

Eukaryotic cell lines

Policy information about cell lines

Cell line source(s)	MDA-MB-231 human breast cancer cells and HeLa human cervical cancer cells were obtained from the American Type Culture Collection (ATCC) in Manassas, Virginia.
Authentication	The cell lines purchased from ATCC were purchased as 'Certified Reference Material' stocks.
Mycoplasma contamination	The cell lines used in this study tested negative for mycoplasma contamination according to the MycoAlert test kit from Lonza.
Commonly misidentified lines (See ICLAC register)	Neither of the cell lines used in this study are listed in the current version (v.9) of the ICLAC register.
